# Co-activation for enhanced K-ion storage in battery anodes

**DOI:** 10.1093/nsr/nwad118

**Published:** 2023-04-25

**Authors:** Yanhong Feng, Yawei Lv, Hongwei Fu, Mihir Parekh, Apparao M Rao, He Wang, Xiaolin Tai, Xianhui Yi, Yue Lin, Jiang Zhou, Bingan Lu

**Affiliations:** School of Physics and Electronics, Hunan University, Changsha 410082, China; School of Physics and Electronics, Hunan University, Changsha 410082, China; School of Physics and Electronics, Hunan University, Changsha 410082, China; Department of Physics and Astronomy, Clemson Nanomaterials Institute, Clemson University, Clemson, SC 29643, USA; Department of Physics and Astronomy, Clemson Nanomaterials Institute, Clemson University, Clemson, SC 29643, USA; Hefei National Research Center for Physical Sciences at the Microscale, University of Science and Technology of China, Hefei 230026, China; Hefei National Research Center for Physical Sciences at the Microscale, University of Science and Technology of China, Hefei 230026, China; School of Physics and Electronics, Hunan University, Changsha 410082, China; Hefei National Research Center for Physical Sciences at the Microscale, University of Science and Technology of China, Hefei 230026, China; School of Materials Science and Engineering, Central South University, Changsha 410083, China; School of Physics and Electronics, Hunan University, Changsha 410082, China; State Key Laboratory of Advanced Design and Manufacturing for Vehicle Body, Hunan University, Changsha 410082, China

**Keywords:** potassium ion batteries, anode, co-activation, high reversible capacity

## Abstract

The relative natural abundance of potassium and potentially high energy density has established potassium-ion batteries as a promising technology for future large-scale global energy storage. However, the anodes’ low capacity and high discharge platform lead to low energy density, which impedes their rapid development. Herein, we present a possible co-activation mechanism between bismuth (Bi) and tin (Sn) that enhances K-ion storage in battery anodes. The co-activated Bi-Sn anode delivered a high capacity of 634 mAh g^–1^, with a discharge plateau as low as 0.35 V, and operated continuously for 500 cycles at a current density of 50 mA g^–1^, with a high Coulombic efficiency of 99.2%. This possible co-activation strategy for high potassium storage may be extended to other Na/Zn/Ca/Mg/Al ion battery technologies, thus providing insights into how to improve their energy storage ability.

## Teaser

A co-activated Bi-Sn material was designed as anode for potassium ion batteries, and the co-activation of Bi and Sn led to the formation of K3Sn, thus overcoming the traditional limit of one Sn atom storing only one K ion.

## INTRODUCTION

Lithium-ion batteries (LIBs) are prevalent in modern society [1–3]. However, limited global lithium resources and safety concerns remain significant barriers to meeting the ever-increasing global need for grid-scale storage [4–6]. As an alternative to LIBs, potassium-ion batteries (PIBs) are attractive because of their low cost (1.5 wt% of K vs. 0.0017 wt% of Li in the Earth’s crust) and lower standard redox potential (−2.93 V vs. SHE (standard hydrogen electrode)) than Na (−2.71 V vs. SHE) [7–11]. Moreover, high energy density has been a hot topic in the research field [12–16]. For example, Niu *et al.* reported a stable Li-C anode based on mesoporous carbon fibers, enabling a Li metal full cell with an energy density of up to 350–380 Wh kg^–1^ [17]. Wang *et al.* developed a quasi-solid-state Na^+^ capacitor with a high energy density of 168 Wh kg^–1^ by rational design of the carbon-based material and electrolyte [18]. Nevertheless, the carbon anode-based PIBs cannot meet the demand for high energy density due to their inherent limited capacity (graphite anode ∼279 mAh g^–1^) [19–22]. Consequently, there is an urgent need to develop anode materials with both high capacity and low discharge platforms, which could be a promising path for PIB-based higher-energy systems [23–26].

**Figure 1.**
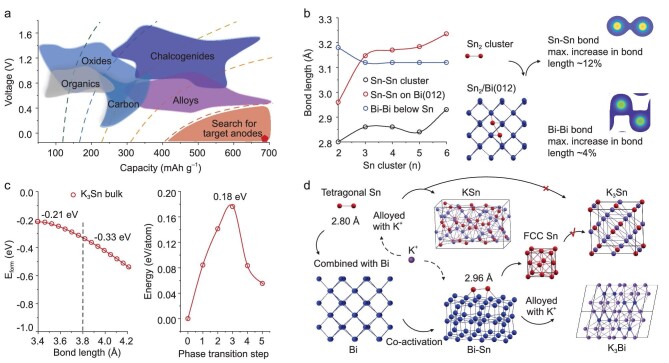
Status of PIB anodes and our design concept for co-activation for enhanced energy storage. (a) Current status and challenges in developing various types of anodes such as carbon, alloys, organics, oxides and chalcogenides in PIBs (red pentagon: K metal). (b) The average Sn–Sn and Bi–Bi bond lengths for isolated Sn_n_ clusters and Sn_n_/Bi(012), and the top view of Sn_2_/Bi(012) and its charge density contours. (c) The formation energies of the K_3_Sn bulk cell during K removal from the structure (left) and the 0.18 eV energy barrier that must be overcome for Sn's phase transition (right). The non-monotonic curves are deduced from the Broyden-Fletcher-Goldfarb-Shanno (BFGS) convergence algorithm, and the activated bond lengths (∼12% increase) of the fully optimized values were used. (d) A schematic illustrating the potassiation products formed due to the beneficial co-activation (red: Sn atom; blue: Bi atom; purple: K atom).

Various materials, such as carbon, alloys, oxides, organics and chalcogenides, have been reported as PIB anodes (Fig. 1a and Supplementary Table S1) [27–31]. Depending on their respective reaction mechanisms, they are classified as intercalation-type, conversion-type and alloy-type anodes [32–34]. Conventional intercalation-type anodes generally have a low capacity (<350 mAh g^–1^), and conversion-type anodes exhibit a high discharge platform (>0.8 V), neither of which are conducive to high energy density storage [35]. In contrast, alloy-type anodes (Bi, Sb, Sn, P, etc.) are attractive due to their relatively low discharge potential and high capacity [36,37]. In particular, Bi and Sn are ideal PIB anode materials due to their cost efficiency, eco-friendliness and high electronic conductivity [38,39]. One Bi atom can transfer three electrons during alloying to form K_3_Bi, providing a theoretical capacity of 385 mAh g^–1^ [40]. Yet the traditional view is that metal Sn anodes transfer at most one electron during alloying to form KSn [41–45], which has a theoretical capacity (226 mAh g^–1^) much lower than that of LIBs (Li_4.4_Sn, 993 mAh g^–1^) [46]. If one Sn atom could enable multiple electron transfers during alloying with multiple potassiums, as with LIBs, the energy density could be increased substantially. Yet there are no studies or reports on this so far. Low energy density is also a common challenge for metal ion batteries (MIBs) with large ionic radii, such as in PIBs, where no anodes with high-capacity low-voltage discharge platforms are available.

In this study, a co-activated Bi-Sn potassium anode with high-capacity low-voltage discharge platforms is developed, which may solve Sn's previous limitations in storing K. The possible co-activation mechanism between Bi and Sn reduced the formation energy for alloying products, which enhanced the K-ions’ reaction kinetics and promoted Sn's multi-electron storage capability. The co-activated Bi-Sn potassium anode is distinct from the reported alloying anodes such as BiSb and FeSb [47–50], which are formed by mixing two homogeneously (atomic-level) dispersed metals [51–53]. While the Bi and Sn crystals form the Bi-Sn composite anodes, they grow alternating intertwined domains along the optimal interfaces that ensure better contacts between domains under Bi^3+^ and Sn^2+^ rapidly reducing conditions, i.e. the co-activated Bi-Sn is a homogenous (nanoscale-level) mixture of Bi and Sn with activated interfaces. As a result, the co-activated Bi-Sn anodes in PIBs exhibit a high capacity of 634 mAh g^–1^ and stable (>500) cycling, with a low discharge plateau of 0.35 V. We also demonstrate that the co-activation concept is valid for other metals, such as Bi and Ge. For instance, the Bi_0.5_-Ge anode exhibited enhanced energy-storage properties for Na ions. These findings illustrate that the co-activation concept that decreased the formation energy of the alloy phase and enhanced metal ion energy storage may be universal and can be extended to other MIBs, such as Zn/Ca/Mg/Al.

## RESULTS AND DISCUSSION

### Designing the Bi-Sn co-activation strategy

Density functional theory (DFT) calculations investigated Bi/Sn co-activation and its potential for potassium storage. Isolated Sn_n_ clusters, Sn_n_/Bi, Bi_n_/Sn composite structures and K_m_Sn_n_ bulk cells were optimized using the Broyden-Fletcher-Goldfarb-Shanno (BFGS) procedure. The results suggested that the Sn_n_ clusters become more stable with an increase in the number of Sn atoms (Supplementary Fig. S1). Configurations of Sn_n_/Bi(012) indicated the formation of Sn-Bi bonds between the Sn_n_ cluster and Bi(012) (Supplementary Fig. S2). Furthermore, the average Sn–Sn and Bi–Bi bond lengths in Sn_n_/Bi(012) were larger than those of the isolated Sn_n_ clusters and Bi(012) surface (3.05 Å) (Fig. 1b), respectively. The maximum increase in bond lengths was 12% and 4%. If we simulate Bi_n_ clusters adsorbed on the Sn(200) surface, the maximum increase in the Sn–Sn and Bi–Bi bond lengths is 10% and 8%, respectively (Supplementary Fig. S3), implying that the Sn and Bi are co-activated, and the Sn activation is more pronounced in co-activated Bi-Sn.

Furthermore, based on the deformation charge density summation in a perpendicular direction to the Bi plane, a ∼0.27 eV charge transfers from the Sn_2_ cluster to the Bi surface in the Sn_2_/Bi(012) (Supplementary Fig. S4a) and produces repulsive dipoles in Sn_2_, leading to increased Sn–Sn bond length and the weakening of the electrostatic interaction [54]. The same occurs with a 0.13 eV charge transfer from the Bi_2_ clusters to the Sn surface in the Bi_2_/Sn(200) (Supplementary Fig. S4b). This is probably because the electronegativity difference between Bi and Sn is not as great, and the large surface-to-volume ratio weakens the electronegativity of the Bi_2_ cluster. In other words, regardless of the Sn_2_/Bi(012) or Bi_2_/Sn(200) configuration, the charge is transferred from the cluster to the surface, leading to the repulsion within the Sn–Sn and Bi–Bi bonds and weakening of electrostatic interactions, thus forming the co-activated interface.

More importantly, the co-activation significantly decreases the charge density in the Sn–Sn bond, and the Bi–Bi bond below, in Sn_2_/Bi(012) (Supplementary Fig. S5). Combining the projected density of states (PDOS), the confined eigenstates of strong metallic Sn–Sn are dispersed in energy, showing the activation effect. When the activated Sn adsorbs K, the K–Sn bond increases from 3.55 Å to 3.70 Å (Supplementary Fig. S6), very close to the 3.84 Å of the K–Sn bond in K_3_Sn, increasing the probability of K_3_Sn formation by activation. Meanwhile, DFT calculations show that the Bi–Bi eigenstates in Bi_2_/Sn(200) also disperse in energy, as in the case of Sn–Sn in Sn_2_/Bi(012) (Supplementary Fig. S7).

Therefore, we conjecture that this co-activation could contribute to energy storage. To explore the K ions storage forms, the K_3_Sn bulk cell was constructed and optimized (Supplementary Fig. S8). The BFGS method simulated the Sn cluster evolution as a function of K atom removal. The formation energy of the K_3_Sn bulk cell decreased from −0.21 to −0.33 eV/atom (Fig. 1c) after the bond length activation, tremendously favoring the formation of the K_3_Sn structure. Besides the energy lowering, another reason for the possible formation of K_3_Sn is the phase transition of Sn (after the co-activation) from the tetragonal to the face-centered cubic (FCC) phase because the Sn occupies the FCC positions in the K_3_Sn crystal. Using a set of linear transition models, an energy barrier of 0.18 eV/atom was obtained for the tetragonal to the FCC phase transition, and the Sn–Sn bond lengths were found to increase from 3.12 to 3.39 Å (Fig. 1c and Supplementary Fig. S9). Thus, the bond length activation is very beneficial for this phase transition and may facilitate the formation of K_3_Sn.

Additionally, there may be a cascading mechanism for co-activation. DFT calculations showed that the bond length of the Sn–Sn cluster on the K_3_Bi surface increased from 2.80 Å to 2.96 Å (Supplementary Fig. S10a and b), and that of the Bi–Bi cluster on the K_3_Sn surface increased from 2.64 Å to 2.82 Å (Supplementary Fig. S10c and d). This means that the K_3_Bi and K_3_Sn formed continuously activate Sn and Bi, i.e. the co-activation was continuous during the reaction.

Briefly, the co-activation decreases the formation energy of alloying products, promotes the phase transition from tetragonal Sn to FCC Sn, and may enhance the formation of K_3_Sn. Therefore, we predict that with co-activation, the traditional limitation of the Sn atom to store one K may be surpassed while forming K_3_Bi to realize enhanced energy storage (Fig. 1d).

### Investigating Bi-Sn co-activation interface formation

**Figure 2.**
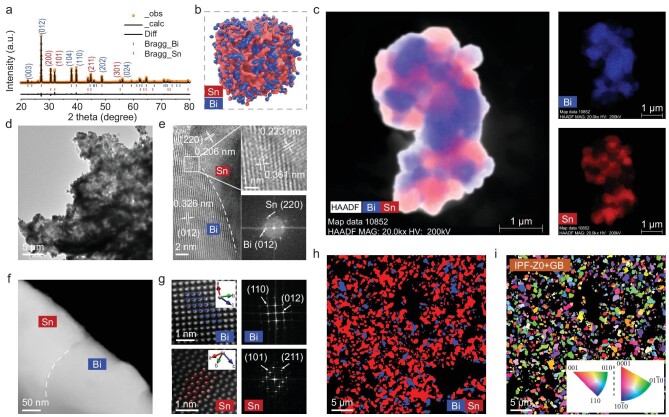
Formation and visual structural characterization of co-activation Bi-Sn anodes. (a) XRD pattern. (b) Structural model. (c) Volume rendering of a tomographic reconstruction and its EDS-STEM mapping. (d and f) TEM images. (e) HRTEM images and the corresponding FFT patterns. (g) Atomic-resolution HAADF-STEM images and FFT patterns of Bi and Sn. (h) EBSD map of phase distribution. (i) Inverse pole figure.

Benefiting from our theoretical calculations, a novel Bi-Sn composite was prepared for realizing high-performance PIB anodes as an experimental verification for the possible co-activation mechanism. The Bi-Sn and reference samples (Supplementary Fig. S11) (Bi : Sn 1 : 0, Bi : Sn 0 : 1, Bi : Sn 1 : 1, Bi : Sn 1.5 : 1, Bi : Sn 0.5 : 1, abbreviated as ‘Bi, Sn, Bi-Sn, Bi_1.5_-Sn, Bi_0.5_-Sn’) were synthesized from Bi(NO_3_)_3_·5H_2_O and SnCl_2_·2H_2_O as sources of Bi and Sn, and C_6_H_5_Na_3_O_7_ as a chelator, following reduction with NaHB_4_ and sedimentation with NH_4_Cl. The digital photographs and scanning electron microscopy (SEM) images of Bi-Sn reveal its three-dimensional (3D) morphology formed by randomly distributed channels containing nanostructured branches (Supplementary Fig. S12a and b). Nanostructured branches of different diameters are evident in transmission electron microscopy (TEM) images (Fig. 2d), and interconnect to form a network of channels with abundant pores and active sites. In contrast, the morphology of Bi and Sn prepared using the same synthesis method (Supplementary Figs S13 and S14) is quite different from Bi-Sn, showing typical nanosheets and microparticles, respectively. Notably, there are no Bi flakes, micron-sized Sn particles or their agglomeration in the Bi-Sn composite (Supplementary Fig. S12c) because Bi and Sn achieve nanoscale dispersion and form rich active Bi-Sn interfaces. As a result, Bi-Sn has a more stable structure, and its 3D structure could promote better electrolyte infiltration, facilitate rapid electron/ion transport, and buffer the volume expansion of the discharge process compared to the sheet and microparticle structure [55–57]. An X-ray diffraction (XRD) pattern and structural refinement were performed on the designed Bi-Sn composite (Fig. 2a), and no BiSn alloy peaks were present. The sharp XRD peaks suggest good crystallinity and match well with those of Bi (PDF#44-1246) and Sn (PDF#04-0673), indicating that Bi^3+^ and Sn^2+^ are fully reduced to Bi and Sn metals and coexist in the composite. The reference samples showed typical peaks corresponding to Bi and Sn (Supplementary Fig. S15). The calculated lattice parameters via the structure refinement are *a* = *b* = 4.54 Å, *c* = 11.87 Å for Bi, and *a* = *b* = 5.84 Å, *c* = 3.19 Å for Sn, which are consistent with the experimental data (Supplementary Table S2).

The TEM image (Fig. 2f) and energy-dispersive X-ray spectroscopy (EDS) mapping (Supplementary Fig. S16) show the intersection of the Bi and Sn phases. High-resolution transmission electron microscopy (HRTEM) clearly showed the lattice crossing at the intersection of the two phases (Fig. 2e). The (012) crystal plane lattice of Bi increases from 0.326 nm to 0.361 nm, and the (220) plane lattice of Sn increases from 0.206 nm to 0.223 nm, indicating that the two phases were co-activated, providing excellent room for the intercalation of large K ions, which are consistent with the calculated results. The inductively coupled plasma emission spectrometer (ICP) revealed Bi_1.03_-Sn as the stoichiometric composition of the prepared Bi-Sn samples, which is very close to the precursor composition of 1 : 1 (Supplementary Table S3).

Additionally, the atomic phases of the two in Bi-Sn samples were analyzed using high-angle annular dark-field scanning transmission electron microscopy (HAADF-STEM, Fig. 2g). The Bi and the Sn matched their corresponding crystallographic models well. To further probe the internal structure of Bi-Sn, we performed HAADF-STEM tomography. Volume tomographic reconstruction of Bi-Sn (Supplementary Fig. S17 and Movie S1) and a single longitudinal section extracted from the volume (Supplementary Fig. S18) showed a uniform distribution of Bi and Sn with a high degree of interfacial contact. These interfacial contacts provide significant active sites for K ions to interact with (Fig. 2c). In addition, Brunauer-Emmett-Teller (BET) revealed a specific surface area of 631.6 m^2^ g^–1^ for the Bi-Sn material (Supplementary Fig. S19a), and the pore size distribution is dominated by mesopores of 2–11 nm that can promote electrolyte transport and buffer volume expansion effectively. Moreover, the mesopores could enable rapid electron transfer to accelerate the reaction kinetics of K ions (Fig. 2b and Supplementary Fig. S19b). The chemical properties of the Bi-Sn samples were also investigated by X-ray photoelectron spectroscopy (XPS), which showed typical spectral signatures for Bi and Sn (Supplementary Fig. S20). The presence of Bi_2_O_3_ 4f_7/2_ (158.68 eV) and Bi_2_O_3_ 4f_5/2_ (163.98 eV) is due to the surface oxidization of the sample when exposed to air [58].

Furthermore, the microstructure and crystallographic orientation of the Bi-Sn samples were analyzed using electron backscatter diffraction (EBSD). The SEM-transmission Kikuchi diffraction (SEM-TKD) pattern of the analyzed region shows thier relatively granular material texture further confirmed the nature of Bi-Sn samples (Fig. 2h), with Sn phase and Bi phase alternatingly distributed in the matrix [59]. The inverse pole figure (IPF) of X, Y and Z orientation indicates that the as-prepared Bi-Sn materials achieved random nanoscale dispersion and relatively uniform grain orientations (Fig. 2i and Supplementary Fig. S21). This uniform orientation property is attributed to the transformation of metal ions into metallic phases during the reduction process, thus forming phase interfaces that benefit the co-activation of the two phases.

### Demonstrating high-performance K batteries

**Figure 3.**
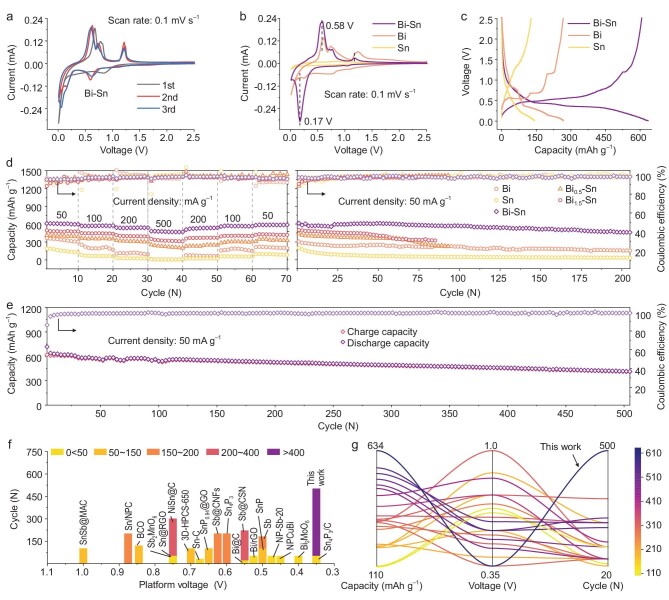
The co-activated Bi-Sn anodes showed enhanced electrochemical properties for K ions. (a) CV curves of a Bi-Sn anode in the first three cycles. (b) CV curves of Bi, Sn and Bi-Sn anodes in their stable cycle. (c) The charge/discharge curves of Bi, Sn and Bi-Sn anodes in their stable cycle. (d) Comparison of rate capacities (left) and cycling stabilities (right) of Bi, Sn, Bi_0.5_-Sn, Bi_1.5_-Sn and Bi-Sn anodes (excluding the cell activation process). (e) Long cycling performance of the Bi-Sn anode. (f and g) Capacity, voltage and cycling comparison of the Bi-Sn anode with the previously reported alloy-type anodes for PIBs (current density was not more than 200 mA g^–1^).

Benefiting from the described specific nature of the Bi-Sn structure, we evaluated the electrochemical properties of Bi-Sn anodes. A potassium foil was used as the counter electrode and 3 M potassium bis(fluorosulfonyl)imide (KFSI) in 1,2-Dimethoxyethane (DME) as the electrolyte (abbreviated as ‘3 M KFSI’). The electrochemical behavior of the Bi-Sn anode was evaluated by cyclic voltammetry (CV) at a scanning speed of 0.1 mV s^–1^ (Fig. 3a). During the cathodic scan for the first three cycles, the broad peak at 0.5–1.0 V (vs. K^+^/K) is due to the formation of the solid electrolyte interface (SEI), while a small shoulder at ∼0.17 V is due to the K ions alloying with metal Bi or Sn in the Bi-Sn anode. Three strong peaks, present at 0.5–1.5 V in the anodic scan, correspond to the dealloying reactions. The CV curves for the fourth, fifth and sixth cycles are highly coherent (Supplementary Fig. S22), showing excellent reversibility and stability. Their charge/discharge platforms (Supplementary Fig. S23a and b) are consistent with the corresponding CV data. We also noted that the CV and charge/discharge curves for the first three cycles of the Bi-Sn anode are significantly different to the last three cycles. This is due to the Bi-Sn anode with a high specific surface area; the formation of SEI is incomplete in the first cycle and continues during the next two cycles. Another possible reason is that the stresses generated by volume change during discharging (i) cause structural reorganization and optimization of the alternating intertwined domains in the Bi-Sn anode, thus exposing more interfacial contacts (forming more SEI) and enhancing the activation effect (which can be viewed as the electrode activation process), and as a result (ii) promote the cascading reaction, i.e. the K_3_Bi and K_3_Sn formed during the reaction can continue activation of Sn and Bi (Supplementary Fig. S10) to achieve holistic co-activation. Thus, interface co-activation may be an entry point that contributes to the cascading co-activation and the reorganization/optimization of Bi-Sn structure during the reaction. Furthermore, the stable CV curves for the Bi, Sn and Bi-Sn anodes at a scan rate of 0.1 mV s^–1^ show that the Bi-Sn anode has a lower alloying/dealloying potential compared to the Bi, Sn anodes (Fig. 3b). What is exciting is that the average discharge platform of the Bi-Sn anode is as low as 0.35 V, and the discharge capacity is as high as 634 mAh g^–1^ (Fig. 3c, including the capacity provided by the combination of Bi-Sn alloying reaction, conductive agent Super P (Supplementary Fig. S27), and nanomaterial surface adsorption (high voltage portion of >0.8 V)), which is higher than the capacities of 266 mAh g^–1^ and 142 mAh g^–1^ provided by the Bi and Sn anodes, respectively. These electrochemical properties, viz., low potential and high capacity, are essential for realizing high-energy-density PIBs.

After the fourth discharge/charge activation cycle, the K ions diffusion coefficients (*D*_K_) were measured using the galvanostatic intermittent titration technique (GITT) for the Bi-Sn and Bi anodes (Supplementary Fig. S24a and b). The cells were discharged/charged at 50 mA g^–1^ for 10 min and then left open-circuit for 60 min to allow complete relaxation to quasi-equilibrium potential. The Bi-Sn anode exhibited a higher diffusion coefficient (*D*_K_, 10^–12^∼10^–8^ cm^2^ s^–1^) during potassiation/depotassiation compared to the Bi anode (*D*_K_, 10^–13^∼10^–10^ cm^2^ s^–1^) (Supplementary Fig. S24c and d), suggesting that the co-activation of Bi and Sn in the Bi-Sn anode allows for an orderly and rapid diffusion of K ions [60].

Rate performances and cycling stability (Fig. 3d) were collected for five anodes, and their charge/discharge profiles showed high similarity (Supplementary Fig. S23c and d). Competitively, the Bi-Sn electrode exhibited reversible capacities of ca. 612, 572, 542 and 484 mAh g^–1^ at 50 to 500 mA g^–1^; corresponding capacity retention was 79%. When returned to 50 mA g^–1^, a reversible capacity of 589 mAh g^–1^ was recovered with a capacity recovery rate of 96% (Supplementary Table S4). By contrast, the capacity retentions and recovery rates of the other four reference anodes were less than satisfactory when the current density was increased 10 times. Similarly, the Bi-Sn anode exhibited the best cycle stability, with a capacity of 463 mAh g^–1^ after 200 cycles. The Bi_0.5_-Sn and Bi_1.5_-Sn anodes failed after operating for less than 100 cycles. This may be because the unbalanced ratios lead to agglomeration (Supplementary Fig. S25), providing fewer active interfaces and, therefore, weaker co-activation. The agglomeration also leads to severe volume expansion and electrode damage, accelerating cell failure. Additionally, the mixed Bi and Sn anode formed by simply grinding Bi powder and Sn powder with 1 : 1 mole ratio shows fewer active interfaces (Supplementary Fig. S26, possibly because they are present next to each other without lattice crossing). Their capacity is much lower (∼400 mAh g^–1^) than that provided by the co-activated Bi-Sn anode, suggesting that Bi did not activate Sn in the mixed Bi and Sn anode to enhance capacity. In contrast, the pure Bi and pure Sn anodes exhibited low capacities of 157 and 37 mAh g^–1^ after 200 cycles. All four reference anodes failed to deliver better performances than the Bi-Sn anode. It follows that the interfacial contacts between Bi and Sn are crucial for co-activation. At a ratio of 1 : 1, Bi-Sn composite anodes are formed by the Bi and Sn crystals (i) growing alternating intertwined domains along the optimal interfaces that ensure better contacts between domains under Bi^3+^ and Sn^2+^ rapidly reducing conditions, (ii) suppressing the respective aggregation of Bi and Sn, and (iii) achieving nanoscale dispersion of co-activated interfaces.

Moreover, the Bi-Sn anode exhibited long-cycle performance and could still provide a high capacity of 417 mAh g^–1^ after 500 cycles with a high Coulombic efficiency of 99.2% (Fig. 3e; the Coulombic efficiencies for the first five cycles were 60.5%, 72.9%, 68.4%, 86.4% and 92.5%, respectively). Here, a capacity of 160–180 mAh g^–1^ at a current density of 50 mA g^–1^ was measured for the conductive carbon (Super P) anode (Supplementary Fig. S27). To the best of our knowledge, compared with previously reported potassium-ion alloy-type anodes such as Bi, Sb, Sn and P, and their alloy phases (Fig. 3f and g), the Bi-Sn anode developed in this work is highly competitive in terms of high capacity, long cycle life and low discharge potential (Supplementary Table S5).

### Studying the potassiation mechanism of Bi–Sn anodes

**Figure 4.**
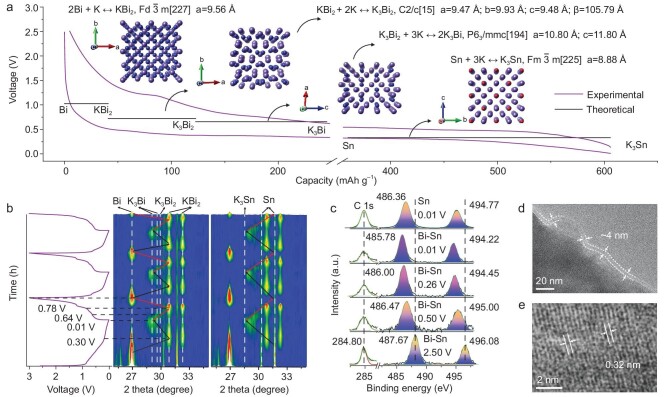
Investigation of the reaction products during potassium storage in a co-activation Bi-Sn anode. (a) DFT-calculated equilibrium voltages of the charge-discharge process and its phase changes. (b) Contour plot of the *in situ* XRD study of the Bi-Sn anode during the first three cycles at 300 mA g^–1^. (c) XPS Sn 3d spectra of Sn at the cut-off voltage of 0.01 V and Bi-Sn anodes at different discharge states of the fourth cycle. (d and e) Cryo-TEM images of SEI nanostructure and K_3_Sn nanocrystals with long-range-ordered lattices.

To unravel the mystery of their extraordinary capacity, *in situ* XRD, semi-*in situ* XPS and cryogenic-transmission electron microscopy (cryo-TEM) combined with DFT were used to investigate the potassium storage mechanism in Bi-Sn anodes alloying/dealloying with K ions. The equilibrium potential of the charge-discharge reaction at stable cycles was determined through DFT simulations (Fig. 4a) [61]. The theoretical stoichiometric compositions of K-Bi and K–Sn matched well with those measured experimentally at each potential (the breakpoint at X-axis can be considered as the capacity provided by Super P). The reaction mechanism of the Bi-Sn anode is summarized as:


\begin{eqnarray*} &&{\rm{Bi}} \leftrightarrow {\rm{KB}}{{\rm{i}}}_2 \leftrightarrow {{\rm{K}}}_3{\rm{B}}{{\rm{i}}}_2 \leftrightarrow {{\rm{K}}}_3{\rm{Bi,}}\\ &&{\rm{Sn}}({\rm{Tetragonal}}) \to {\rm{Sn}}({\rm{FCC}}) \leftrightarrow {{\rm{K}}}_3{\rm{Sn.}} \end{eqnarray*}

After stable cycling, we confirmed the existence of FCC Sn by HRTEM in the charged state of the Bi-Sn anode. The lattice spacing of 0.238 nm for FCC Sn corresponds well to the DFT’s crystalline model (Supplementary Fig. S28). This lattice spacing differs from tetragonal Sn, which has a relatively large lattice spacing of 0.306 nm, signifying Sn's phase transition and promoting K_3_Sn formation.

The above-described evolution during charge-discharge is further supported by *in situ* XRD patterns (Fig. 4b). During discharging, the peak intensity corresponding to Bi and Sn in the Bi-Sn anode gradually decreases, and the peaks corresponding to the alloy phases with K ions appear. During charging, the successive diminishing of peak intensity of each alloying phase is accompanied by the gradual recovery of the Bi and Sn peaks, implying a fully reversible process. Specifically, during discharging, the Bi and Sn peak intensities fade, and the KBi_2_ (PDF#03-0698) peaks appear at 30.9° and 32.2° [62]. When the discharge is nearly complete (∼0.01 V), the KBi_2_ peaks intensity diminishes, and the K_3_Bi (PDF#04-0642) peaks at 28.9° and 29.7° appear along with a peak at 28.5°, tentatively assigned to the K_3_Sn. The peaks located at ∼25.5° in the initial stage of *in situ* XRD are assigned to Bi_2_O_3_, which is also consistent with our XPS data (Supplementary Fig. S20). The disappearance of Bi_2_O_3_ during the subsequent charge/discharge process is due to the following reaction of the oxide on the surface of the material in the initial discharged state: Bi_2_O_3_ + 6K^+^ + 6e^−^ → 2Bi + 3K_2_O and 2Bi + 6K^+^ + 6e^−^ ↔ 2K_3_Bi. It is known that Bi_2_O_3_ + 6K^+^ + 6e^−^ → 2Bi + 3K_2_O is irreversible as K is more easily oxidized than Bi, and the next reaction is a reversible reaction of alloying/dealloying between Bi and K_3_Bi. Also, the content of Bi_2_O_3_ is very small, and the contribution of Bi_2_O_3_ will disappear after the electrode activation. Bi_2_O_3_ contributes to the electrochemical reaction but less so relative to Bi-Sn.

Note that K_3_Sn is an absent phase in the available databases. Therefore, we have synthesized different ratios of potassium-tin alloys (Supplementary Fig. S29a, prepared by heating and mixing K metal and Sn powder, see Methods for details). At a ratio of 1 : 2, the XRD pattern shows two peaks at 31.5° and 32.9°, which correspond to K_4_Sn_23_ (JCPDS no.04-8647) [63]. When the ratio reaches 1 : 1, peaks at 29.2°, 30.2° and 32.0° are attributed to the KSn phase [64]. Further increasing the ratio to 3 : 1, two strong peaks appear at 24.2° and 34.2°, corresponding to metal K (PDF#01-0500), in addition to the K_4_S_23_ and KSn peaks, indicating a complete reaction of Sn with K and an excess of K. More interestingly, a new peak appears at 28.5° when the ratio is 3 : 1, corresponding to the K_3_Sn peak in the fully discharged state of the Bi-Sn anode (the peaks present at 29.7° and 31.0° are K_3_Bi and KBi_2_ phases, respectively). Moreover, the XRD reflections of the Bi-Sn anode in the discharged state correspond well to the DFT-predicted peaks for K_3_Sn. The peaks at 28.5°, 40.7°, 50.3°, 58.9° and 66.4° correspond to the (220), (400), (422), (440) and (620) crystal planes of K_3_Sn, respectively (Supplementary Fig. S29b). The DFT-predicted XRD pattern resembled the XRD pattern of the experimentally synthesized K_3_Sn (Supplementary Fig. S29c), further validating our previous conjecture that the activated Sn in the Bi-Sn anode may be alloyed with K to form the K_3_Sn phase.

We also performed semi-*in situ* XPS on Bi-Sn anodes at different discharge depths and compared the results to those of the fully discharged state of the Sn anode (Fig. 4c). The bond energies of Sn decreased to 486.36 eV (Sn3d 5/2) and 494.77 eV (Sn3d 3/2) when the Sn anode was fully discharged, and the KSn alloy phase was detected by XRD (Supplementary Fig. S30). But no KSn alloy peaks were detected for the Bi-Sn anode discharged to 0.01 V. In comparison, the binding energy of Sn decreased to 485.78 eV (Sn3d 5/2) and 494.22 eV (Sn3d 3/2) when Bi-Sn was discharged to 0.01 V (Fig. 4c). (It should be mentioned that the XPS studies were repeated to check for the reproducibility of small shifts in the Sn peaks (Supplementary Fig. S31), which possibly provides evidence for alloy formation other than KSn; please see Methods for details). This is because on the K metal side, K − e^−^ → K^+^ occurs during discharge, and K^+^ and e^−^ move through the electrolyte and external circuit, respectively, to reach the Bi-Sn electrode for alloying reactions. With increasing discharge depth, more and more K^+^ move towards the Bi-Sn side, leading to a gradual increase in the electron cloud density around Sn, decreasing the binding energy (peaks shift left). It can be speculated that the Bi-Sn alloys with K, and the KSn is not formed. Instead, the activated Sn atom in Bi-Sn stores more than one K ion, further hinting at Sn's multi-electron energy-storage mechanism in the Bi-Sn anode.

In addition, HAADF-EDS mapping, inductive coupled plasma emission spectrometer (ICP) and selected area electron diffraction (SAED) were used to investigate the chemical composition of the alloy phases formed at the Bi-Sn anode in the discharged state. The K, Bi and Sn elements were detected when discharged to 0.01 V (Supplementary Fig. S32). The ratio of K/Bi-Sn atomic percentages remained constant at ∼3.38 (Supplementary Fig. S33), which is close to 3 : 1, consistent with the results of the ICP (Supplementary Table S6) and SAED (Supplementary Fig. S34) data. The SAED revealed the multi-crystalline nature of K_3_Bi and K_3_Sn, further indicating that the final potassiation products under co-activation might be K_3_Bi and K_3_Sn. Meanwhile, using cryo-TEM, the alloy phases for K_3_Sn and K_3_Bi in the fully discharged state were also examined (see Methods for details). We found that the sample's surface was homogeneously coated with a layer of amorphous SEI, which plays a crucial role in the stable cycling of the electrode (Fig. 4d and Supplementary Fig. S35). Moreover, the ∼0.320 nm lattice spacing corresponds to the (220) crystal plane of K_3_Sn (Fig. 4e), while the ∼0.303 nm lattice spacing corresponds to the (110) crystal plane of K_3_Bi, which is consistent with the *in situ* XRD and DFT calculations.

### Applying and expanding the co-activation strategy

**Figure 5.**
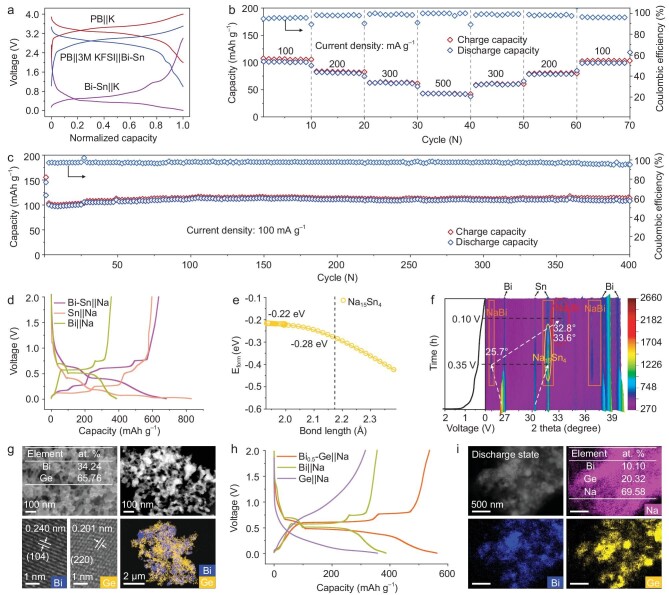
Co-activation energy storage in full cells and the extension of this to SIBs. (a) Typical charge/discharge curves of the half cells of a PB cathode, Bi-Sn anode, and their full cells. (b) Rate performance at various current densities of full cells. (c) Cycling performance of a full cell. (d) Charge/discharge curves for Bi-Sn, Bi and Sn anodes in SIBs. (e) The formation energy of the Na_15_Sn_4_ clusters during Na removal from the structure. (f) Contour plot of the *in situ* XRD results of the Bi-Sn anode in SIBs at 100 mA g^–1^. (g) SEM, HAADF-STEM, HRTEM and EDS mapping of the Bi_0.5_-Ge sample. (h) Charge/discharge curves for Bi_0.5_-Ge, Bi and Ge anodes in SIBs. (i) HAADF-STEM and EDS mapping of a Bi_0.5_-Ge sodium anode in the discharged state (all scale bars = 500 nm).

With practical applications in mind, we have synthesized Prussian blue (PB) based on reported work (see Methods for details) [65]. The XRD pattern (Supplementary Fig. S36a) of the synthesized PB cathode corresponds well to the characteristic XRD pattern of Fe_4_(Fe(CN)_6_)_3_ (PDF#73-0687). It shows a bulk morphology (Supplementary Fig. S36b), indicating the successful synthesis of PB. The PB cathode (K‖PB half-cell) has a capacity of ∼110 mAh g^–1^ when tested at a current density of 100 mA g^–1^ in the voltage range of 2.0–4.0 V (Supplementary Fig. S37). Next, the PB cathode was paired with a deep pre-potassiated Bi-Sn anode and assembled into a potassium-ion full cell (soft pack and CR2032 type) with 3 M KFSI as electrolyte (Supplementary Fig. S38). The full cell has a high average voltage, consistent with the difference between the PB cathode's discharge voltage and the Bi-Sn anode's charge voltage (Fig. 5a). More significantly, the full cell exhibited high rate capacities (based on the total mass of the cathode and anode) of 102, 82, 63 and 43 mAh g^–1^ at 100 to 500 mA g^–1^ (Fig. 5b). When the current density was reduced to 100 mA g^–1^, the capacity returned to 99 mAh g^–1^ with a recovery rate of 97%, showing excellent reversibility. In addition, the cycling stability is outstanding, with a high capacity of 119 mAh g^–1^ at 100 mA g^–1^ and a capacity decay rate of only 0.023% per cycle after 400 cycles of continuous operation (Fig. 5c). At the same time, the full cell could power everyday devices, such as an LED panel or a blood glucose or uric acid analyzer, with still plenty of power left after one year of use (Supplementary Fig. S39). Additionally, we tested the full cell consisting of the untreated Bi-Sn anode and the PB cathode. It exhibited an initial charge capacity of ∼37 mAh g^–1^ at 500 mA g^–1^, but the discharge capacity was extremely low and almost absent (Supplementary Fig. S40). The root cause was the potassium deficiency during the initial cycle and its irreversible loss in cycling, which resulted in extremely poor electrochemical performance [66]. Thus, deep pre-potassiation (see Methods for details) is important for improving the performance of full cells [67].

Lastly, to broadly explore the co-activation concept for energy storage, we evaluated the electrochemical performance of SIBs as a testbed. Interestingly, the change of the charging/discharging platforms of the Bi-Sn sodium anode showed a typical evolutionary path of Bi and Sn alloying with Na, respectively (Fig. 5d). And DFT calculations demonstrated that the formation energy of Na_15_Sn_4_ can be decreased from −0.22 to −0.28 eV/atom, which, as in the case of potassium storage, significantly reduces the formation difficulty of the Na_15_Sn_4_ structure (Fig. 5e and Supplementary Fig. S41). With deepening discharge, the *in situ* XRD analysis showed that the Bi-Sn anode undergoes sodiation as follows: Bi → NaBi → Na_3_Bi; Sn → Na_15_Sn_4_ (Fig. 5f and Supplementary Fig. S42). The experimental data confirmed that the co-activation mechanism favors the formation of the final Na_3_Bi and Na_15_Sn_4_ products, thus mitigating the formation of other mesophase alloys such as NaSn and Na_9_Sn_4_ [68]. Furthermore, we know that Ge metal can transfer 4.4 electrons just like Sn to form the Li_4.4_Ge alloy, providing a theoretical capacity of up to 1600 mAh g^–1^ [69], whereas at most, one electron can be transferred to form an NaGe alloy that provides a theoretical capacity of 369 mAh g^–1^ [70]. If Ge can react with more than one Na, its performance is bound to be enhanced even more. Therefore, Bi_0.5_-Ge samples were synthesized in the same way to maximize the performance of Ge. As evidenced by the SEM and HAADF-STEM images, the Bi_0.5_-Ge samples exhibited finer cross-linked nanoparticles than the Bi-Sn samples, which could lead to enhanced co-activation (Fig. 5g). The XRD pattern showed good crystallinity of Bi (PDF# 44-1246) and Ge (PDF# 04-0545) in Bi_0.5_-Ge (Supplementary Fig. S43b). With lattice spacings of 0.240 nm and 0.201 nm corresponding to the (104) and (220) crystal planes of Bi and Ge, respectively, the two elements are distributed uniformly (Fig. 5g and Supplementary Fig. S43a). More importantly, the Bi_0.5_-Ge sodium anode also demonstrated enhanced energy storage properties over the Bi and Ge anodes, with a high capacity of 562 mAh g^–1^ (Fig. 5h). When discharged to 0.01 V, higher Na content (Fig. 5i and Supplementary Fig. S44), in addition to Bi and Ge elements, was detected in the Bi_0.5_-Ge electrode, consistent with ICP results (Supplementary Table S7). The high sodium content indicates that Bi_0.5_-Ge may have enhanced energy storage capacity for Na. Furthermore, the 1D scans of the *in situ* XRD study showed that Bi ↔ NaBi ↔ Na_3_Bi occurs during the charging/discharging process. The peaks of Ge weaken during discharging and recover during charging (orange frame), but no Na-Ge alloy peak appears (Supplementary Fig. S45), which indicates that the Na-Ge alloy formed during discharge may be an amorphous phase. SAED also confirmed this, and Na_3_Bi crystal's spots were detected in fully discharged Bi_0.5_-Ge samples, while the whole sample showed amorphous characteristics (Supplementary Fig. S46). Collectively, the data presented in this study for PIBs and SIBs confirm that co-activation of anodes is feasible in reducing the formation energy of alloy products and enhancing metal ions for energy storage. In the future, we aim to expand and verify this possible co-activation strategy to gain new insights for other (Zn/Ca/Mg/Al) energy-storage systems.

## CONCLUSION

In summary, we designed a co-activated Bi-Sn electrode material and evaluated its performance as PIB anodes. The 3D mesoporous structure could alleviate the volume expansion during potassiation/depotassiation and facilitate rapid electron transfer. The co-activation of Bi and Sn may lead to K_3_Sn formation for enhancing K-ion storage in battery anodes. This study also found a low (0.35 V) discharge plateau for the Bi-Sn anode, which enabled PIBs to achieve a high capacity of 634 mAh g^–1^ and a cycle life of up to 500 cycles. Notably, full cells consisting of a PB cathode and a deep pre-potassiated Bi-Sn anode exhibited stable cycling and good rate performance. These exceptional electrochemical properties make Bi-Sn a promising PIB anode. Moreover, we showed that the co-activation approach works well in SIBs with Bi_0.5_-Ge anodes and is effective in reducing the formation energy of the alloy phase and enhancing the energy storage of metal ions. Nonetheless, based on our present data, we still suggest co-activation as a possible mechanism, and the final potassiation product is possibly K_3_Sn, to explain the high capacity. We hope that our work will spur further exploration by energy researchers, with the goal of better understanding the origin of experimentally observed high capacity and promoting the development of high-capacity PIBs and beyond.

## METHODS

### Materials synthesis

#### Preparation of Bi–Sn or Bi_0.5_-Ge composite materials

Taking the preparation of Bi-Sn (1 : 1) as an example, first, 3.87 g C_6_H_5_Na_3_O_7_ was added to 45 mL deionized water and stirred evenly. Then, Bi(NO_3_)_3_·5H_2_O and SnCl_2_·2H_2_O were added to the above solution in a 1 : 1 ratio and stirred to a white suspension. Finally, 1.7 g NaBH_4_ was quickly added under vigorous stirring, and then 4 g NH_4_Cl was added to settle. Aging for more than 6 h and washing in deionized water a few times followed, then freeze-drying to obtain a 3D Bi-Sn composite material. The other four reference materials, Bi-Sn (1 : 0), Bi-Sn (0 : 1), Bi-Sn (0.5 : 1) and Bi-Sn (1.5 : 1), were prepared by adjusting the mole ratio of Bi to Sn. Mixed anodes of Bi and Sn were prepared by simply mixing Bi powder and Sn powder in a 1 : 1 molar ratio and grinding for more than 1 h. The Bi_0.5_-Ge sample was synthesized as above, except that the Sn source was replaced with GeO_2_.

#### Preparation of PB materials

PB material was prepared by a simple co-precipitation method. First, 0.85 g of K_4_Fe(CN)_6_ was dissolved in 320 mL of deionized water and stirred well to obtain solution No. 1, while 1.08 g of FeCl_3_·6H_2_O was dissolved in 80 mL of deionized water and stirred well to obtain solution No. 2. Then, solution No. 2 was added dropwise to solution No. 1 for a co-precipitation reaction under rapid stirring, and the mixed liquid was stirred for 2 h and aged for 24 h. Finally, it was washed with deionized water and alcohol and dried under vacuum at 80°C for 24 h to obtain PB materials.

#### Preparation of potassium-tin alloys

Potassium-tin alloys in different ratios (1 : 2, 1 : 1, 3 : 1) were prepared in an argon-protected glove box. A potassium block was placed in a magnetic boat and heated to a molten state on a heating plate of 150°C. The desired proportion of tin powder was mixed and left to react at 150°C for 2 h to obtain potassium-tin alloys.

### Electrode preparation

The Bi-Sn, Bi_0.5_-Ge anode and PB cathode were prepared as follows: 60 wt% active material, 30 wt% Super P and 10 wt% poly(vinylidene fluoride) (PVDF) were mixed and ground uniformly (∼30 min). N-methylpyrrolidone (NMP) solvent was added and stirred for 8 h to obtain a uniform slurry, which was then coated on the aluminum foil. After vacuum drying the coated foil at 80°C for 12 h, round electrodes (diameter = 12 mm) were prepared for use. The Super P as an anode was prepared directly by mixing and grinding Super P with PVDF at 9 : 1 (mass ratio); the other steps were the same as above. The mass loading for half cells of Bi-Sn, Bi_0.5_-Ge, Super P anodes and the PB cathode was ∼0.7 mg cm^–2^ (active material). For half cells, the N/P ratio was ∼1.5, and the total mass was ∼1.0 mg cm^–2^. The preparation of other reference electrode (Bi_0.5_-Sn and Bi_1.5_-Sn) materials was the same as above.

## Supplementary Material

nwad118_Supplemental_Files
