# Supplementary material for: Co-activation for enhanced K-ion storage in battery anodes
Source: Natl Sci Rev. 2023 Apr 25;10(7):nwad118. doi: 10.1093/nsr/nwad118 (PMC10306327; doi:10.1093/nsr/nwad118)
Supplement: nwad118_Supplemental_Files [file nwad118_supplemental_files.zip › Supplementary data.pdf]

Supplementary information

Co-activation for enhanced K-ion storage in battery anodes

Yanhong Feng[1], Yawei Lv[1], Hongwei Fu[1], Mihir Parekh[2], Apparao M. Rao[2], He Wang[3], Xiaolin Tai[3], Xianhui Yi[1], Yue Lin[3], Jiang Zhou[4] and Bingan Lu[1,5,*]

[1]School of Physics and Electronics, Hunan University, Changsha 410082, China;

[2]Department of Physics and Astronomy, Clemson Nanomaterials Institute, Clemson University, Clemson, SC 29643, USA;

[3]Hefei National Research Center for Physical Sciences at the Microscale, University of Science and Technology of China, Hefei 230026, China;

[4]School of Materials Science and Engineering, Central South University, Changsha 410083, China;

[5]State Key Laboratory of Advanced Design and Manufacturing for Vehicle Body, Hunan University, Changsha 410082, China;

***Corresponding author**. E-mail: luba2012@hnu.edu.cn

The PDF file includes:

Supplementary Fig. S1 to S49

Supplementary Table S1 to S7

References (1-71)

Movie S1

METHODS

Chemicals

Trisodium citrate dihydrate ($C_6H_5Na_3O_7$, 99.99%), sodium borohydride ($NaBH_4$, 99.98%), ammonium chloride (NH_4Cl, 99.9%), bismuth nitrate pentahydrate ($Bi(NO_3)_3 \cdot 5H_2O$, 99.9%), stannous chloride dihydrate ($SnCl_2 \cdot 2H_2O$, 99.9%), potassium hexacyanoferrate(II) ($K_4Fe(CN)_6$, 99.9%), Iron(III) chloride hexahydrate ($FeCl_3 \cdot 6H_2O$, 99.9%), Germanium dioxide (GeO_2, 99.9%), aluminum foil, potassium (K, 99.9%), sodium (Na, 99.9%), bismuth powder (Bi, 99.8%), tin powder (Sn, 99.8%), potassium bis(fluorosulfonyl)imide (KFSI, 99.8%), N-methyl-2-pyrrolidone (NMP, 99.95%), polyvinylidene fluoride (PVDF, average M_w ~ 534,000 by GPC), Sodium hexafluorophosphate ($NaPF_6$, 99.8%), Super P (99%), 1,2-Dimethoxyethane (DME, 99%), ethylene carbonate (EC, 99%), and diethyl carbonate (DEC, 99%) were purchased from Macklin, Sigma or Aladdin. All materials were used directly without further purification.

Electrolyte preparation

Electrolytes of 3M KFSI in DME or 1M $NaPF_6$ in EC/DEC were prepared in conventional ways. Usually, 6.577 g of KFSI was added to 10 mL DME and stirred for 6 h to get 3 M KFSI electrolyte; 1.7 g of $NaPF_6$ was added to a mixture of 5 mL EC and 5 mL DEC and stirred for 6 h to obtain 1M $NaPF_6$ electrolyte.

Electrochemical Measurements

All CR2032-type potassium ion half and full cells were assembled inside a glove box.

For potassium ion half-cells, the potassium foil was used as the counter electrode and Bi-Sn as the working electrode, while the potassium ion full cell used PB as the cathode and Bi-Sn as the anode. The glass fiber paper was used as the separator, and the 3M KFSI (~ 120 μL) was used as the electrolyte. It should be mentioned that before assembling the full cell, the anode was prepotassiated in a half cell. The pre-potassiation method as follows: (i) potassium block was rolled into potassium sheets with a thickness of 0.4 mm using a roller press, the prepared Bi-Sn electrode sheets (12 mm diameter) were attached to the potassium sheets and gently pressed until tight fit and immersed and stored in the electrolyte for the surface pre-potassiation and electrolyte penetration process; (ii) the Bi-Sn electrode (immersed for at least 2 h) was removed from the potassium sheet and assembled with a fresh potassium sheet (14 mm diameter) to form a K||Bi-Sn half-cell for deep pre-potassiation, i.e., discharging the assembled K||Bi-Sn half-cell to 0.001 V at a low current density of 20 mA g^{-1}; (iii) the K||Bi-Sn half-cell is disassembled and the deeply pre-potassiated Bi-Sn electrode was removed and matched with the PB cathode to assemble the PB||Bi-Sn full cell. Pre-potassiation are the important steps for achieving higher energy density and longer cycling life of the full cell. All assembled cells were left to stand 8 h and then tested at a constant current in a 28℃ temperature chamber. The anode and cathode half-cell voltage ranges were 0.01-2.5 V and 2.0-4.0 V, respectively, while the full battery voltage range was 1.0-3.5 V. And for the application of Bi-Sn anode in sodium-ion half-cells, sodium metal was used as a counter electrode, 1M $NaPF_6$ (~ 120 μL) as the electrolyte. The sodium ion half-cell

voltage range was 0.01-2.0 V.

Characterizations

The morphology, nanostructure, and crystallographic information of the fabricated materials were investigated by scanning electron microscopy (SEM, JEOL JSM-6380LV FESEM), transmission electron microscopy (TEM), high-resolution transmission electron microscopy (HRTEM), and high-angle annular dark-field scanning transmission electron microscopy (HAADF-STEM). X-ray diffraction (XRD, Bruker D8 Advanced diffractometer) and x-ray photoelectron spectroscopy (XPS, VG scientific ESCALAB 250) measurements were used to study the crystal structure and chemical composition. Brunauer-Emmett-Teller (BET) analyzed the materials' specific surface area and pore size distribution. *In situ* XRD was performed to analyze the alloying reaction mechanism in-depth. The microstructure and crystal orientation of the samples were analyzed using electron backscatter diffraction (EBSD). Cryogenic-transmission electron microscopy (Cryo-TEM) was used to study the morphology and SEI nanostructure of the discharged alloy compounds. Half-cell and full-cell electrochemical performances were measured using the Neware test system (BTS-CT-3008-TC 5.X). Cyclic voltammetry (CV) was measured using the CHI660 electrochemical workstation.

Calculation Method

The density functional theory (DFT) calculations of the cluster and cell systems were performed using the open-source plane-wave package QUANTUM ESPRESSO [1,2]. The Perdew-Burke-Ernzerhof (PBE) exchange-correlation functionals were used, with

a kinetic energy cut-off of 639 eV for all the atom species [3,4]. The room-temperature Fermi-Dirac smearing was adopted considering the metallicity. The Broyden-Fletcher-Goldfarb-Shanno (BFGS) quasi-Newton algorithm was adopted to release the forces of the system and simulate the evolutions of Sn systems after the potassium release. The maximum trusted ionic displacement was 0.1 Å in each BFGS step. The system structures were fully optimized until the force on each atom, and total energy variations were smaller than 2.6×10^{-3} eV/Å and 1.4×10^{-3} eV [5]. To avoid mirror interactions, a vacuum space of 15 Å was added perpendicular to the Bi plane, and the dipole correction was considered in the charge density calculations [6]. The Brillouin zone k-point sampling was 5 × 5 × 5 for the unit cell calculation, and only the Γ point was considered for the clusters [3]. The linear cell size and atom position transformations provided the phase transition simulation.

The formation energies were calculated according to equation [4]:

$$E_{\mathrm{form}} = \frac{E(\mathrm{K_mSn_n}) - E(\mathrm{Sn_n}) - \mathrm{m} \times E(\mathrm{K})}{\mathrm{m}}$$

where $E(\mathrm{K_mSn_n})$ and $E(\mathrm{Sn_n})$ correspond to the total energies of K_mSn_n and Sn_n clusters; respectively, and E(K) is the energy of a K atom in a vacuum.

The equilibrium potentials were calculated according to equation:

$$U = \frac{E_{K_xM} - xE_K - E_M}{-xe}$$

where x is the number of electrons involved in the formation reactions, U is the equilibrium potential of the reaction, E_{K_xM} (M=Bi or Sn), E_K, and E_M are the computed total energies of K_xM, K, and M at 0 K by DFT, respectively.

Method of GITT test

The assembled K||Bi-Sn and K||Bi cells are left to stand overnight and then tested at a constant current in a 28°C temperature chamber. All cells were discharged/charged four cycles at the current density of 50 mA g^{-1} under the 0.01-2.5 V voltage to finish the electrode activation process. They are then discharged/charged at the same current density for 10 min and left open-circuit for 60 min to allow complete relaxation to quasi-equilibrium potential for running one cycle.

The diffusion coefficient of potassium ions (D_K) of GITT was calculated according to the equation:

$$D_K = \frac{4}{\pi\tau}\left(\frac{n_m V_m}{S}\right)^2\left(\frac{\triangle E_S}{\triangle E_t}\right)^2$$

where n_m, V_m are the mole number and molar volume of the electrode material, S is the contact area of the electrode/electrolyte, τ is the relaxation time, $\triangle E_S$ is the voltage change due to the pulse, and $\triangle E_t$ is the voltage change for constant current charging (discharging).

Method of semi in-situ XPS study

First, the potassium block was rolled out to a thickness of 0.4 mm using a roller press. A circular mold with a diameter of 14 mm was used to cut out a coupon from the potassium foil. Note that the diameter of the potassium coupon should be larger than the diameter of the working electrode (12 mm) to ensure a sufficient reaction. Then, the half-cells are assembled using the potassium coupon as the counter electrode and the Bi-Sn and Sn anodes as the working electrodes. The assembled cells were left to stand for 8 h and subjected to 3 charge/discharge cycles for electrode activation with a voltage window of 0.01-3.0 V. Then the K||Bi-Sn half-cells were discharged to 2.5 V,

0.5 V, 0.26 V, 0.01 V, respectively, and the K||Sn half-cells were discharged to 0.01 V. Finally, the cells were disassembled in a glove box, and the corresponding electrodes were washed several times in DME solvent to remove the surface SEI and residual electrolyte before XPS tests were performed (after the solvent had evaporated completely).

Method for proving K_3Sn, K_3Bi lattice spacing, and the SEI layer

We first performed EDS mapping on the sample under HAADF, found four target areas (marked numbers 1-4 shown in Supplementary Fig. S47), and noted their exact location on the copper grid (Supplementary Fig. S48).

These samples were then transferred and rapidly cooled under liquid nitrogen for cryo-electron microscopy. According to the elemental distributions, the K_3Sn and K_3Bi lattices were taken at locations with low Bi and Sn contents, respectively. The tests in target areas 1-3 proved unsuccessful due to differences in sample thickness and the samples' instability under the electron beam (too thick: the electron beam did not penetrate, too thin: the sample was unstable because of overheating).

Fortunately, the K_3Sn and K_3Bi lattice, and the SEI layer could be imaged in the red and blue frames in target 4 (Supplementary Fig. S49). The 0.320 nm lattice corresponds to the (220) crystal plane of K_3Sn, while the 0.303 nm lattice corresponds to the (110) crystal plane of K_3Bi.

Supplementary Figures

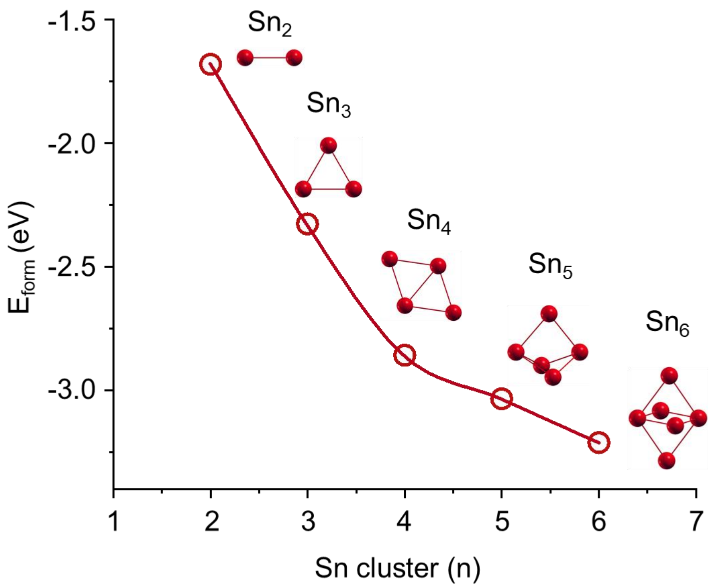

Supplementary Fig. S1. DFT calculations for Sn_n clusters. Formation energies of the Sn_n (n=2-6) clusters (gas phase) and their optimized structures (red: Sn atom).

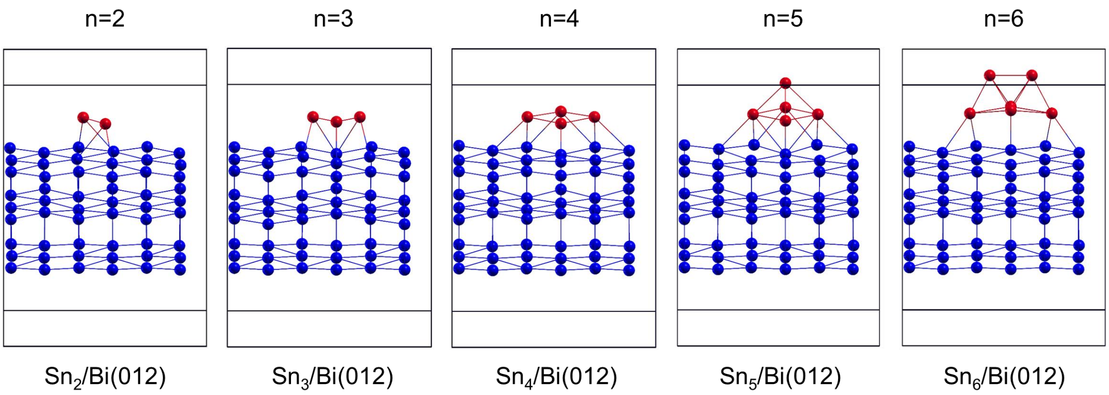

Supplementary Fig. S2. Geometry and stable configurations of Sn_n/Bi(012). Sn_n clusters (n=2-6) adsorbed on (012) crystal plane of Bi (red: Sn atom; blue: Bi atom).

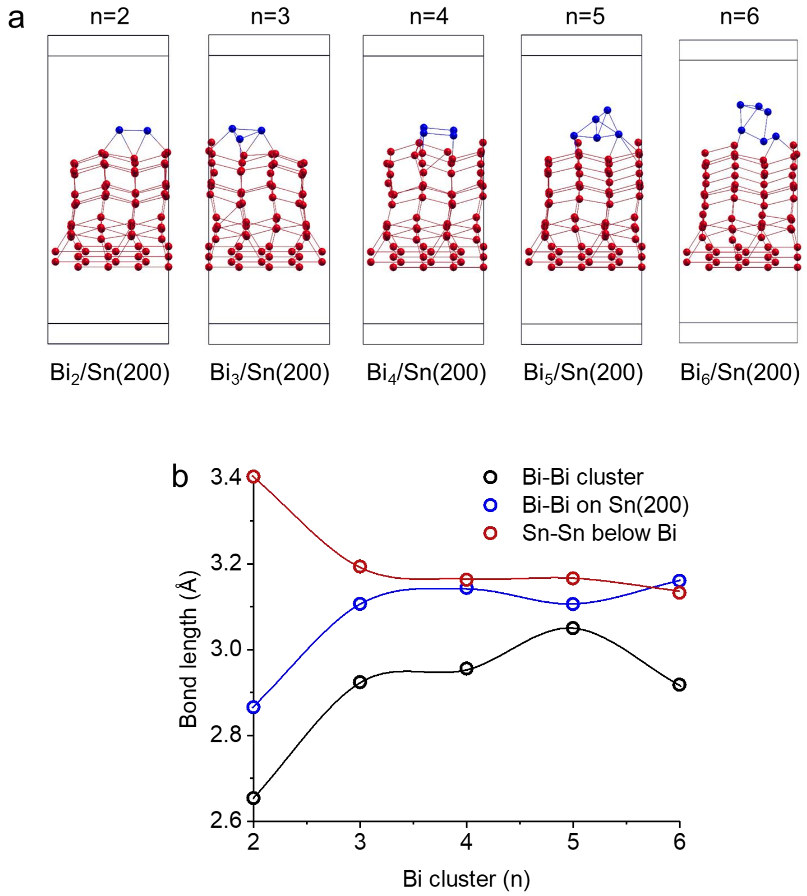

Supplementary Fig. S3. Geometry and stable configurations of Bi_n/Sn(200). (a) Bi_n clusters (n=2-6) adsorbed on (200) crystal plane of Sn. (b) The average Sn-Sn and Bi-Bi bond lengths for isolated Bi_n clusters and Bi_n/Sn(200) (red: Sn atom; blue: Bi atom).

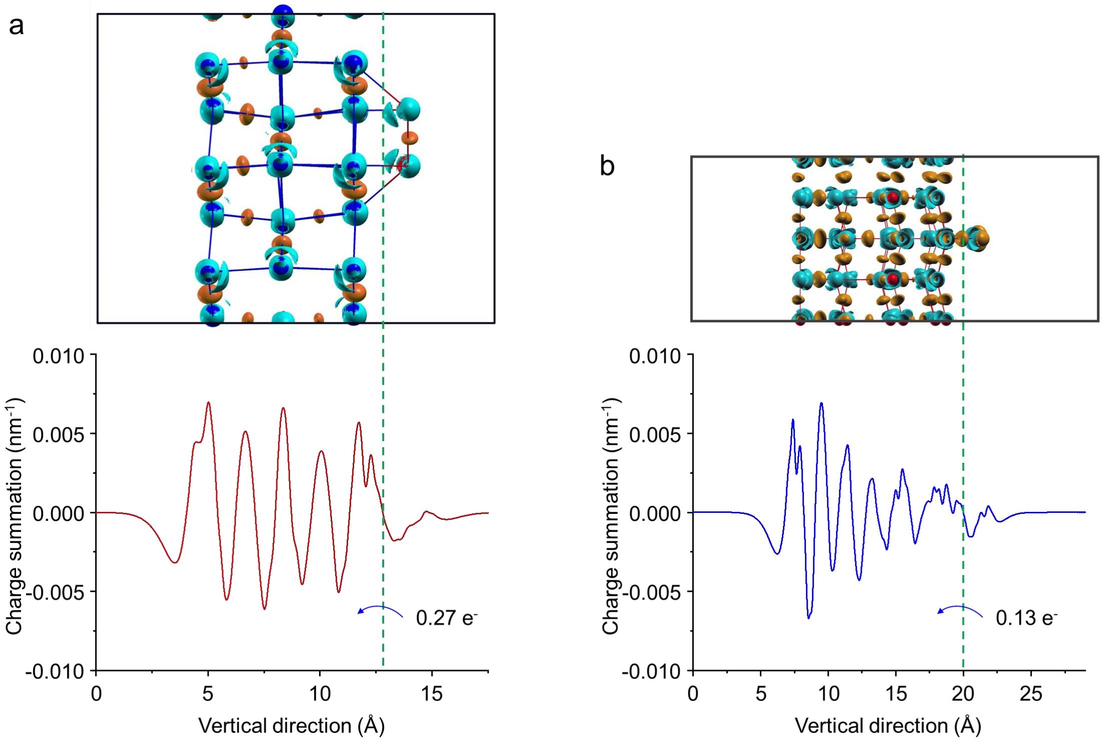

Supplementary Fig. S4. Charge density distribution in Sn_2/Bi(012) (a) and Bi_2/Sn(200) (b). The brown and cyan isosurfaces are electron-rich and electron-deficient, respectively, with values set to 0.004 eV. Their summation along the vertical direction is also shown below, in which the green line separates the Sn_2 (Bi_2) from the Bi(012) (Sn(200)) crystal plane. The dipole correction is employed to restrain the charge transfers between adjacent cells.

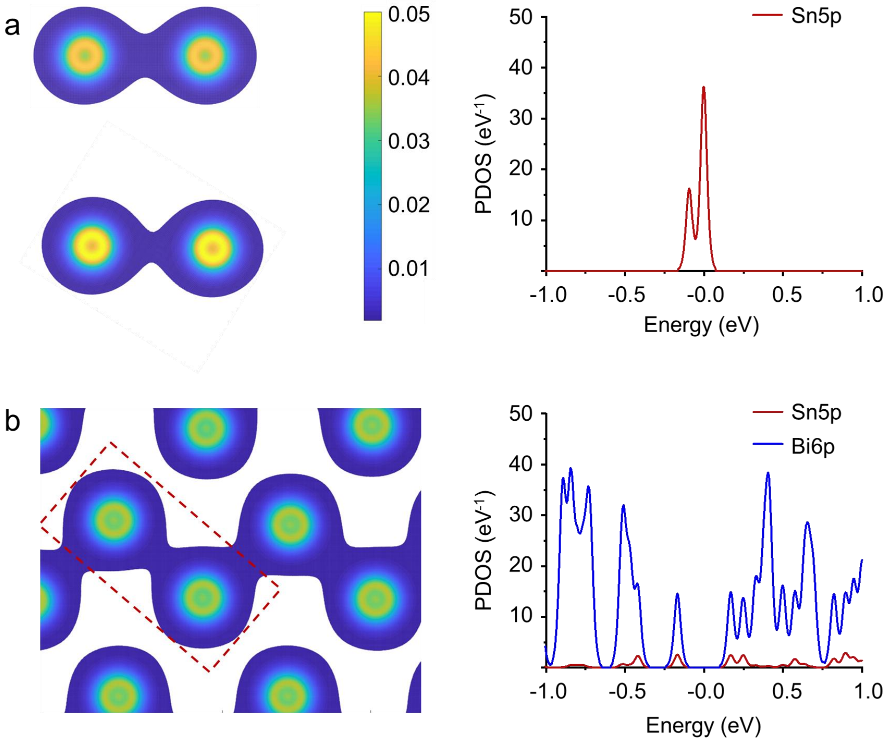

Supplementary Fig. S5. Plots of real-space charge density and energy-space density of states (DOS). Top view of the real-space charge density and energy-space DOS in Sn_2 cluster (a) and Sn_2/Bi(012) (b).

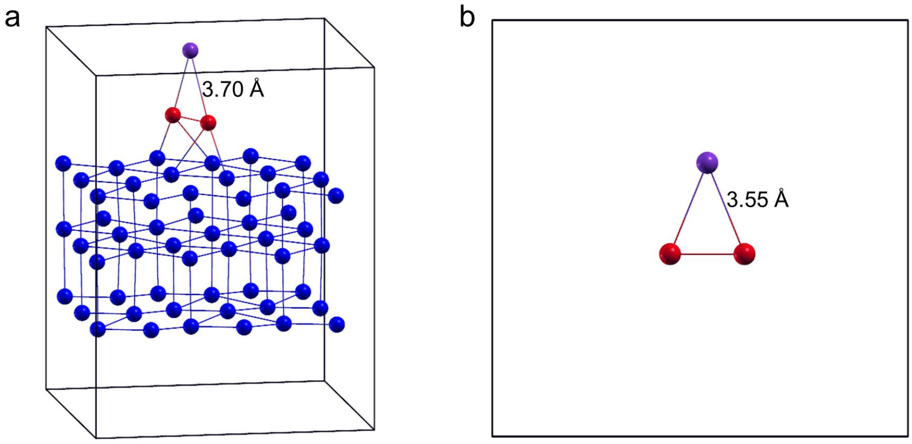

Supplementary Fig. S6. Geometry and stable configurations of K-Sn_2/Bi(012) and K-Sn_2 clusters (isolated) (purple: K; red: Sn atom; blue: Bi atom).

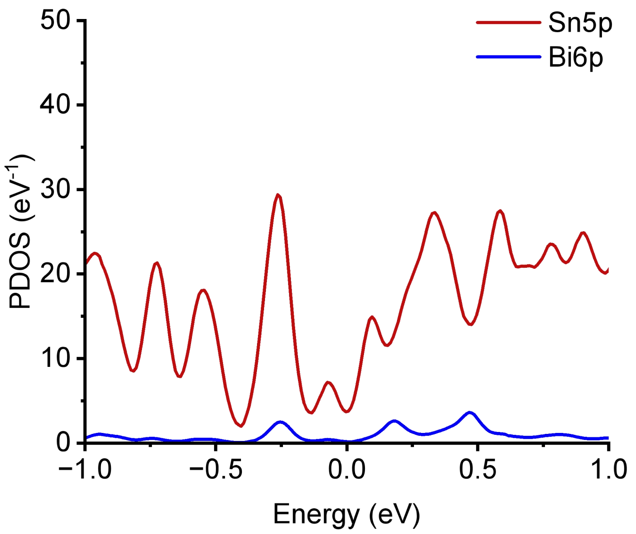

Supplementary Fig. S7. Density of states (DOS) vs. energy for Bi_2/Sn(200).

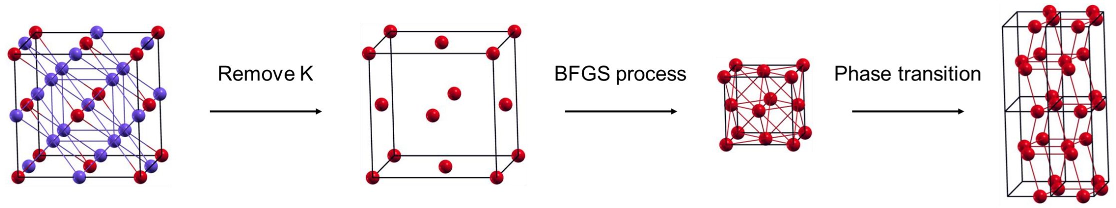

Supplementary Fig. S8. Schematic illustrations of the K_3Sn bulk cell, K-removed Sn cell, and its optimized structure (purple: K atom; red: Sn atom).

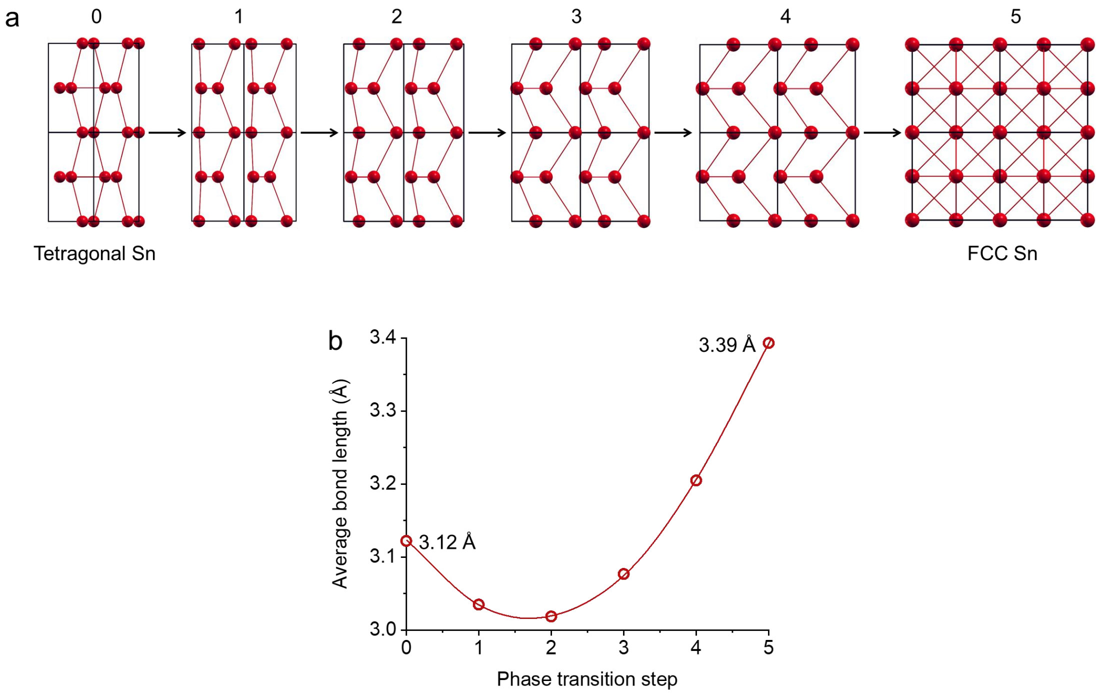

Supplementary Fig. S9. (a) The progression of phase transition from the tetragonal Sn cell to the FCC Sn. (b) Variation of Sn-Sn bond length with phase transition (red: Sn atom).

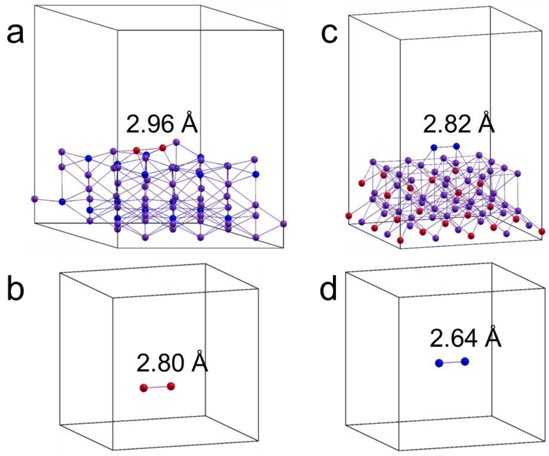

Supplementary Fig. S10. Geometry and stable configurations of Sn_2/K_3Bi(001) (a), isolated Sn_2 clusters (b), Bi_2/K_3Sn(001) (c), and isolated Bi_2 clusters (d) (red: Sn atom; blue: Bi atom).

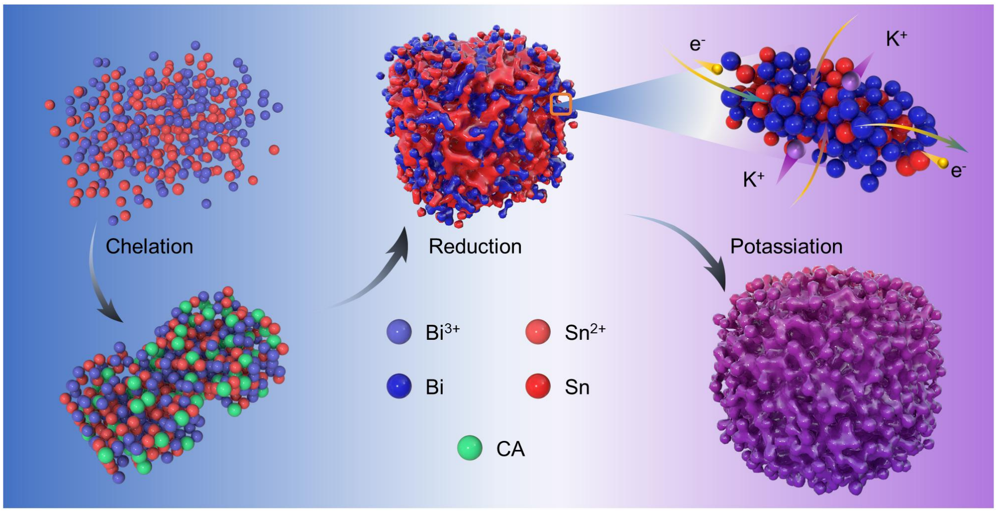

Supplementary Fig. S11. Synthesis and potassium storage in Bi-Sn. Schematic diagram of the synthesis and potassium storage process in Bi-Sn materials (purple: K^+; yellow: e^-).

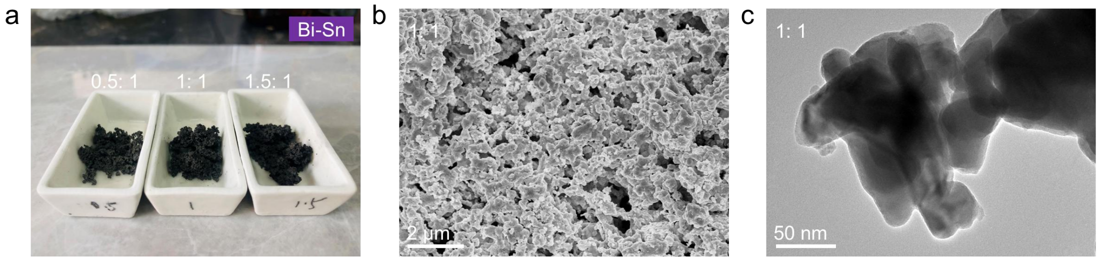

Supplementary Fig. S12. Morphological and structural characterization of the designed Bi-Sn samples. (a) Digital photographs. (b) SEM image. (c) TEM image.

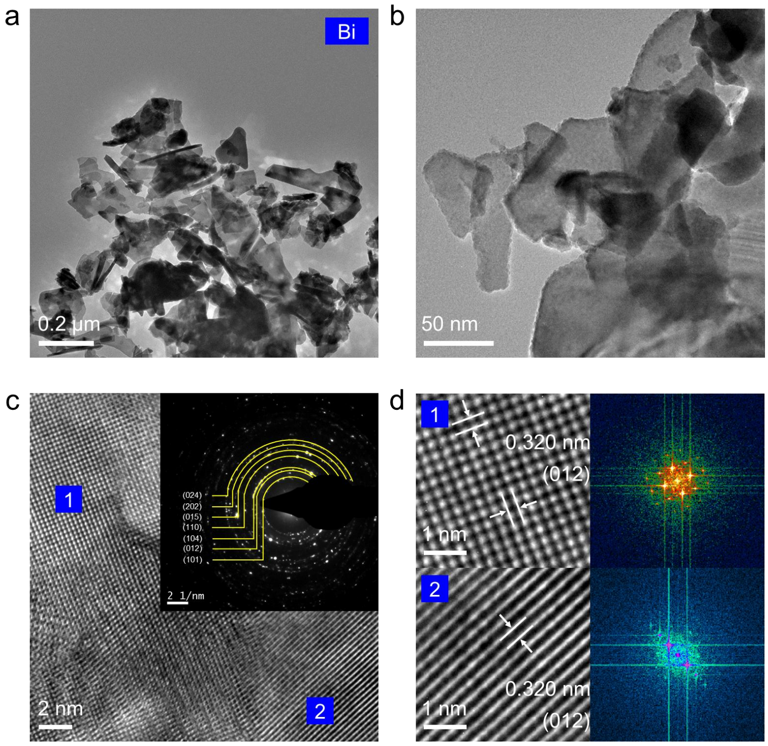

Supplementary Fig. S13. Structural characterization of Bi sample. (a and b) TEM images. (c) HRTEM and its SAED images. (d) HRTEM and its FFT patterns.

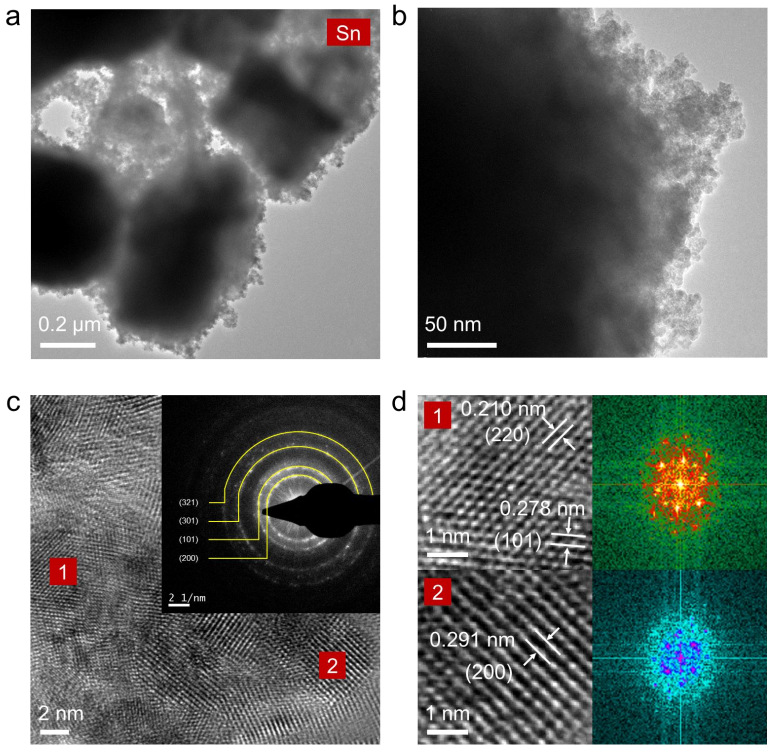

Supplementary Fig. S14. Structural characterization of Sn sample. (a and b) TEM images. (c) HRTEM and its SAED images. (d) HRTEM and its FFT patterns.

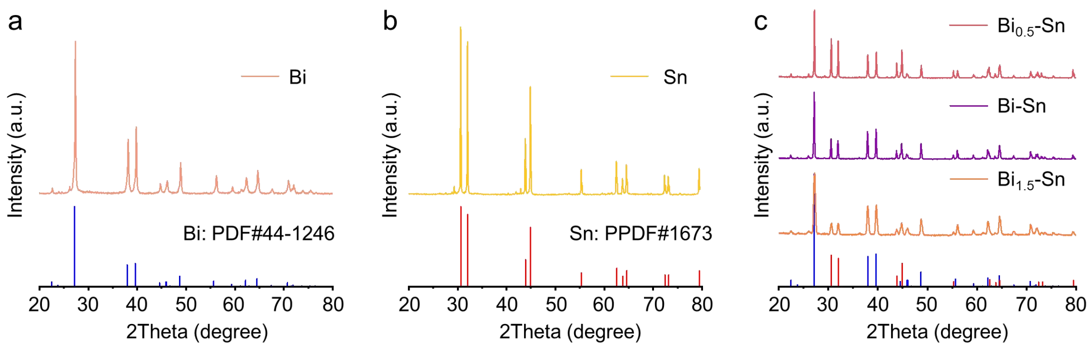

Supplementary Fig. S15. XRD analysis. (a) Bi XRD pattern. (b) Sn XRD pattern. (c) XRD patterns of $Bi_{0.5}$-Sn, Bi-Sn, and $Bi_{1.5}$-Sn.

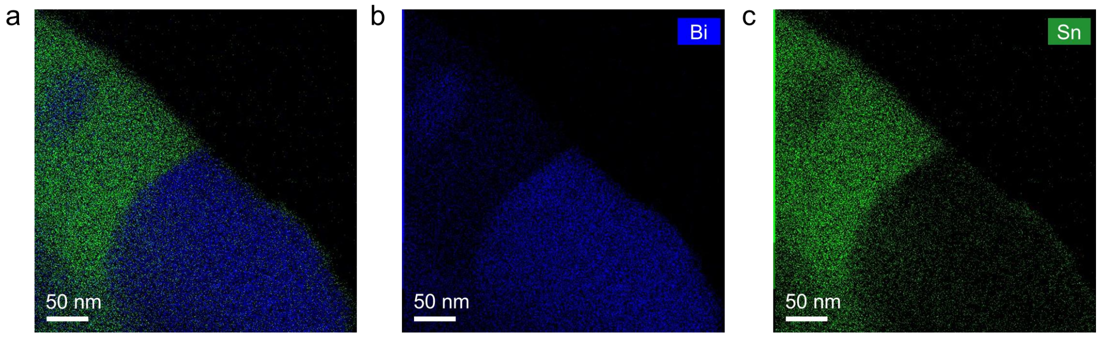

Supplementary Fig. S16. EDS mapping for Bi-Sn. (a) The mapping of the two phases at the boundary. (b) The elemental distribution of Bi. (c) The elemental distribution of Sn.

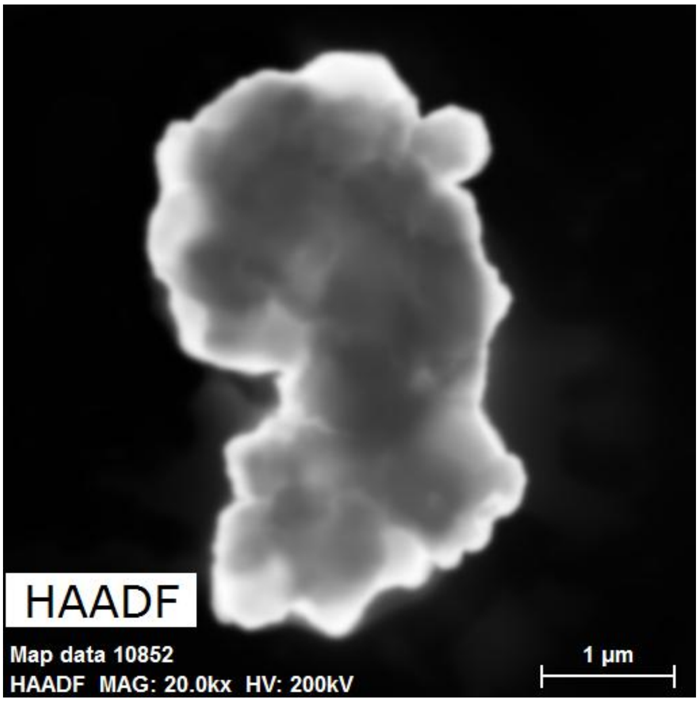

Supplementary Fig. S17. The STEM for volume rendering of tomographic reconstruction.

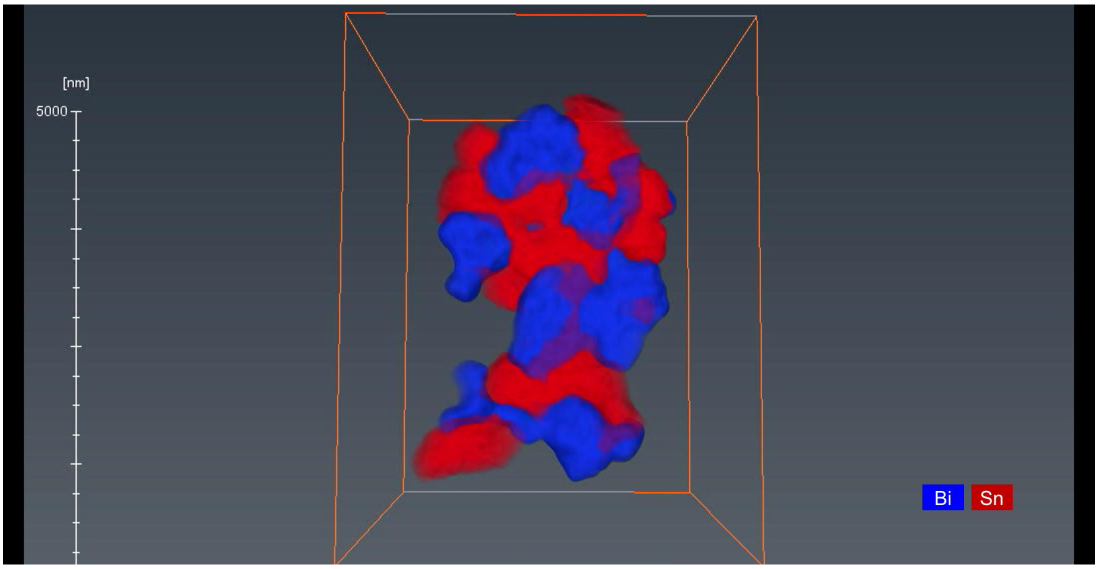

Supplementary Fig. S18. The single longitudinal section from the tomographic reconstruction.

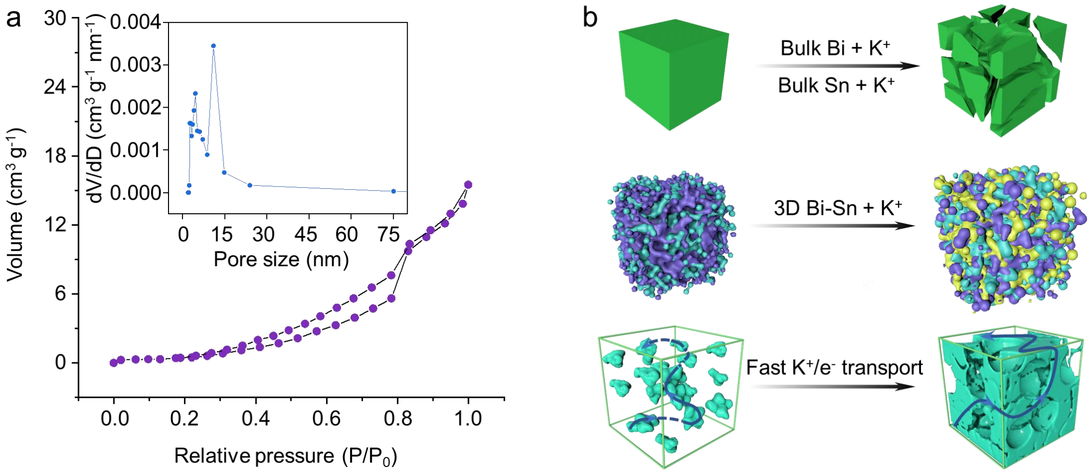

Supplementary Fig. S19. BET tests and structural model of Bi-Sn. (a) BET and pore distribution. (b) The structural advantage over bulk Bi and Sn.

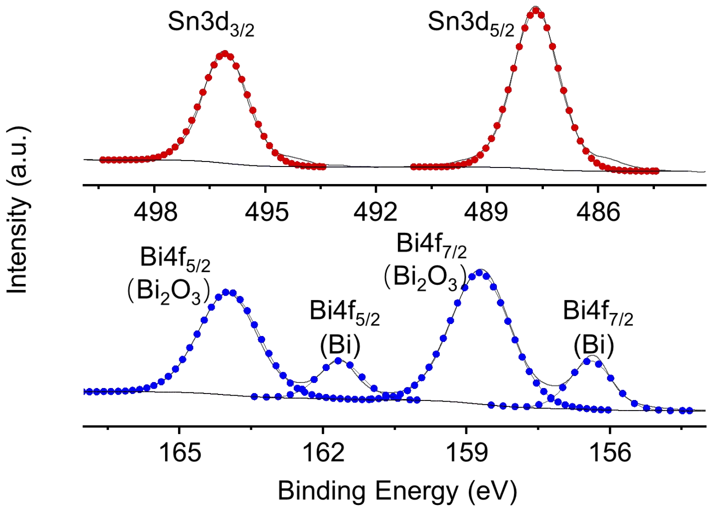

Supplementary Fig. S20. XPS spectral signatures of Bi4f and Sn3d in the Bi-Sn sample.

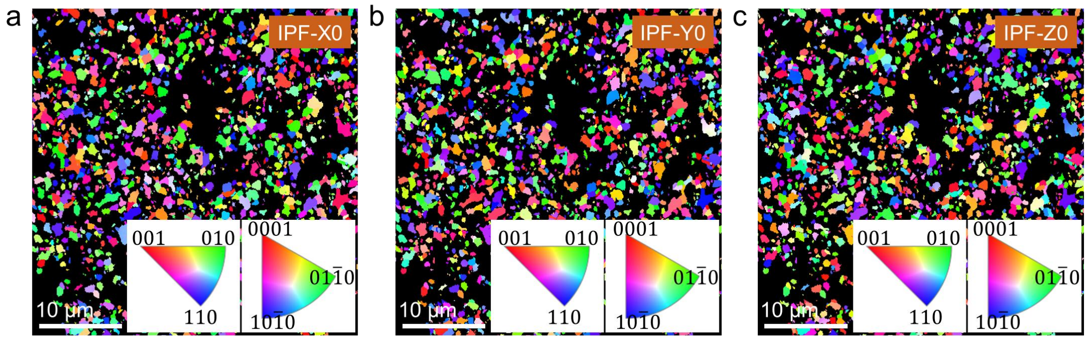

Supplementary Fig. S21. Phase distribution maps and inverse pole figures of the Bi-Sn sample. (a) X direction. (b) Y direction. (c) Z direction.

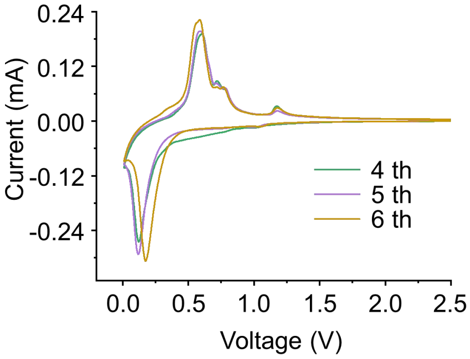

Supplementary Fig. S22. CV curves of Bi-Sn anode in the last three cycles.

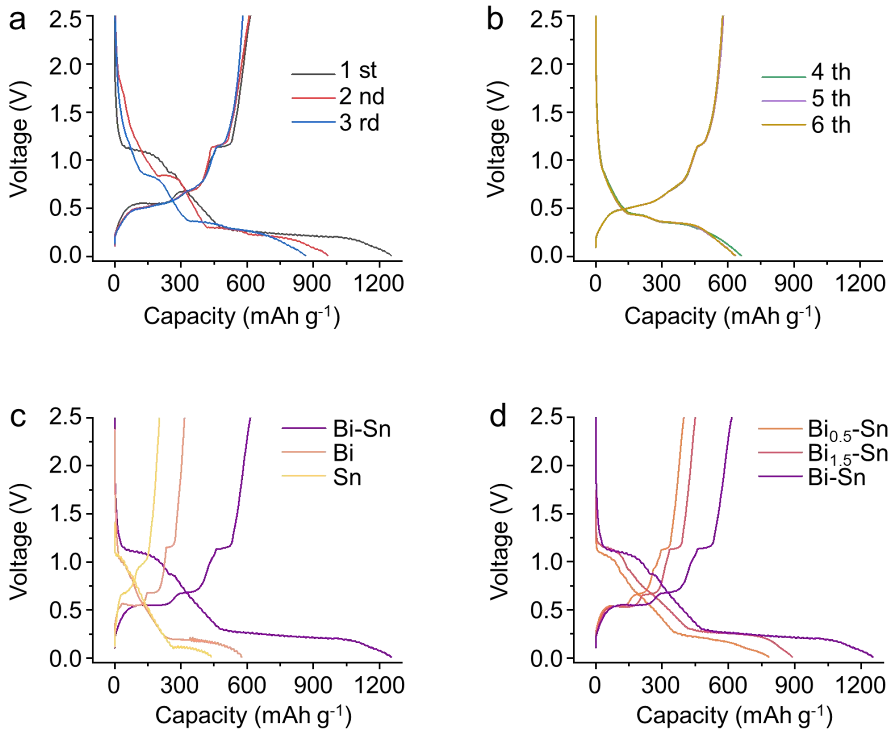

Supplementary Fig. S23. The charge/discharge curves at the current density of 50 mA g^{-1}. Charge/discharge profiles of the first three cycles (a) and the last three cycles (b) of the Bi-Sn anode. (c) Charge/discharge curves of Bi-Sn, Bi, and Sn anodes. (d) Charge/discharge curves of $Bi_{0.5}$-Sn, $Bi_{1.5}$-Sn, and Bi-Sn anodes.

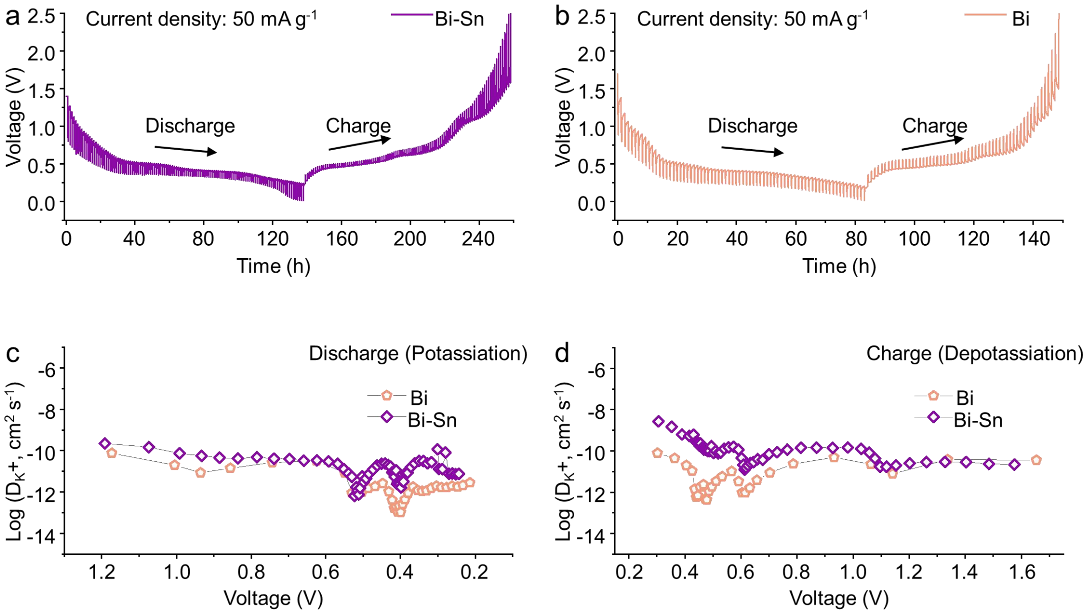

Supplementary Fig. S24. The transient voltage curve versus time for potassiation/depotassiation at 50 mA g^{-1} and the calculated chemical diffusion coefficient D_K. GITT experiment for Bi-Sn anode (a) and Bi anode (b). The D_K of Bi and Bi-Sn anodes during discharging (c) and charging (d) process.

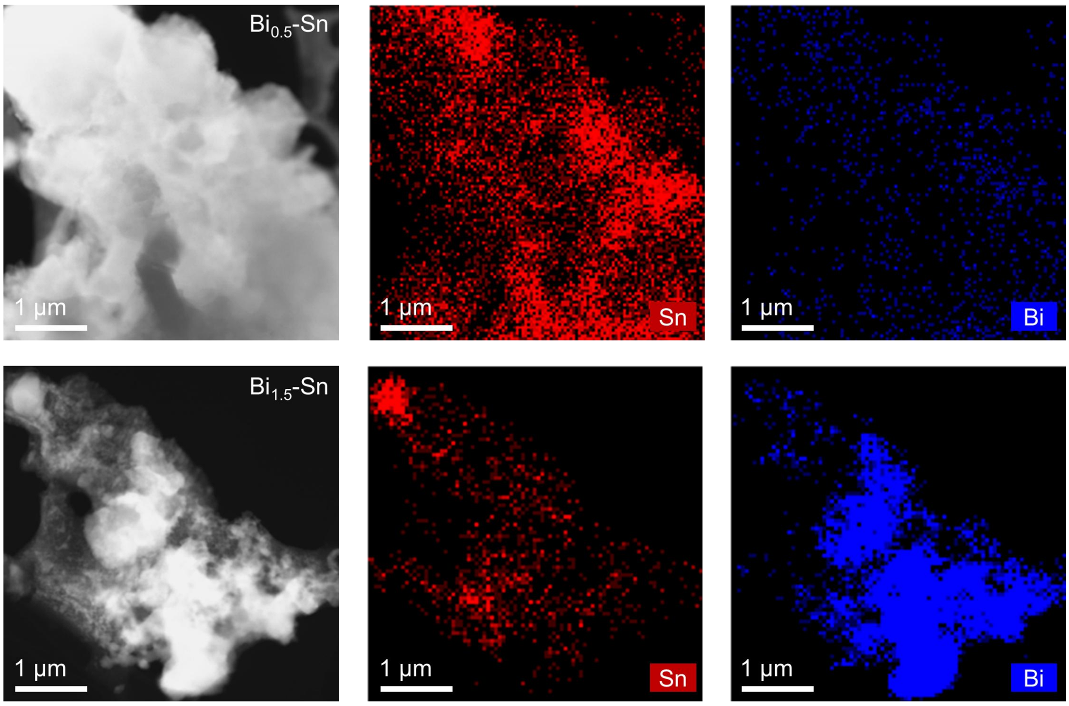

Supplementary Fig. S25. SED mapping of $Bi_{0.5}$-Sn and $Bi_{1.5}$-Sn.

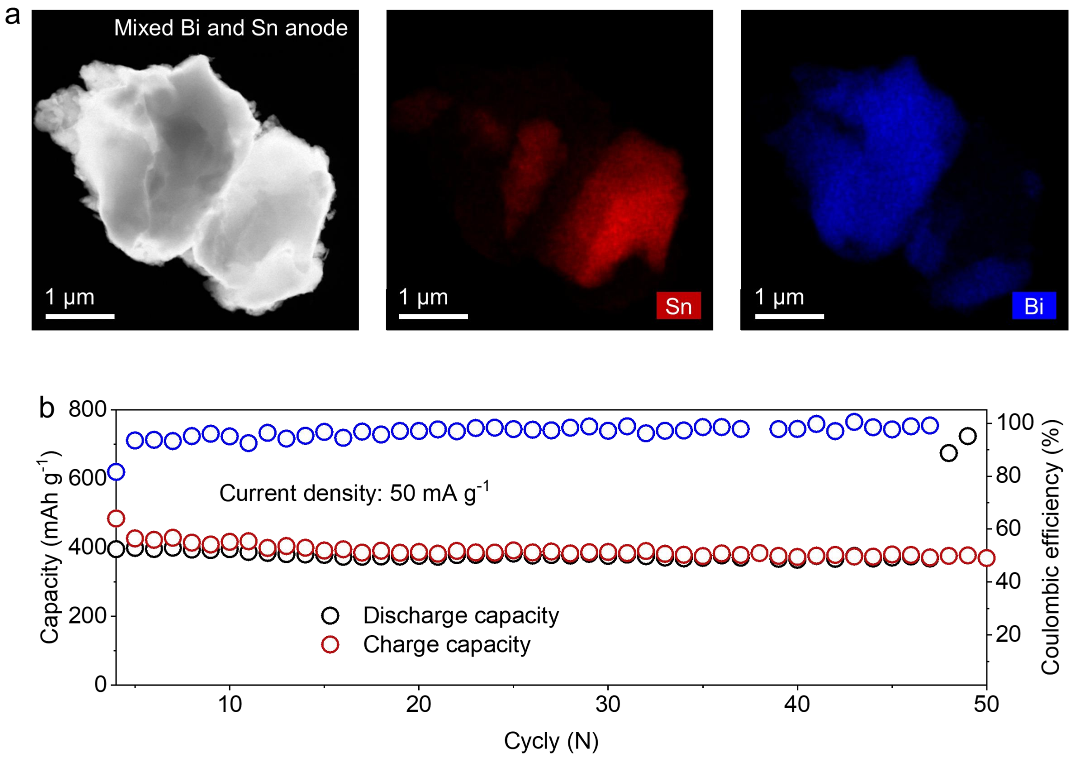

Supplementary Fig. S26. (a) SED-STEM mapping of the mixed Bi and Sn. (b) Cycling and capacity of the mixed Bi and Sn anode.

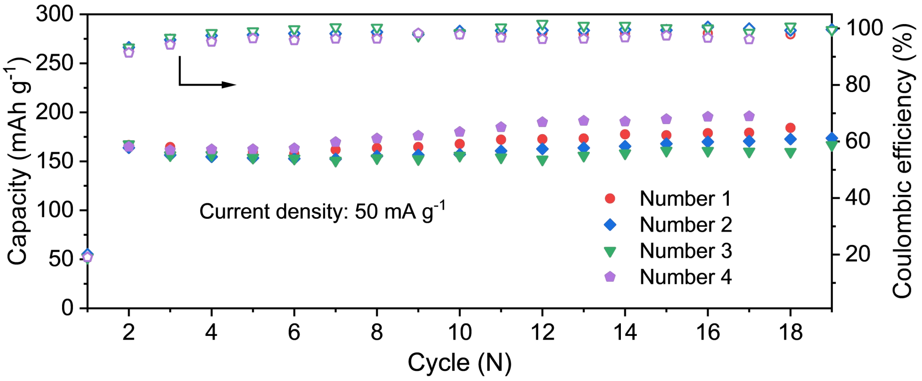

Supplementary Fig. S27. Cycling and capacity of the conductive carbon (Super P).

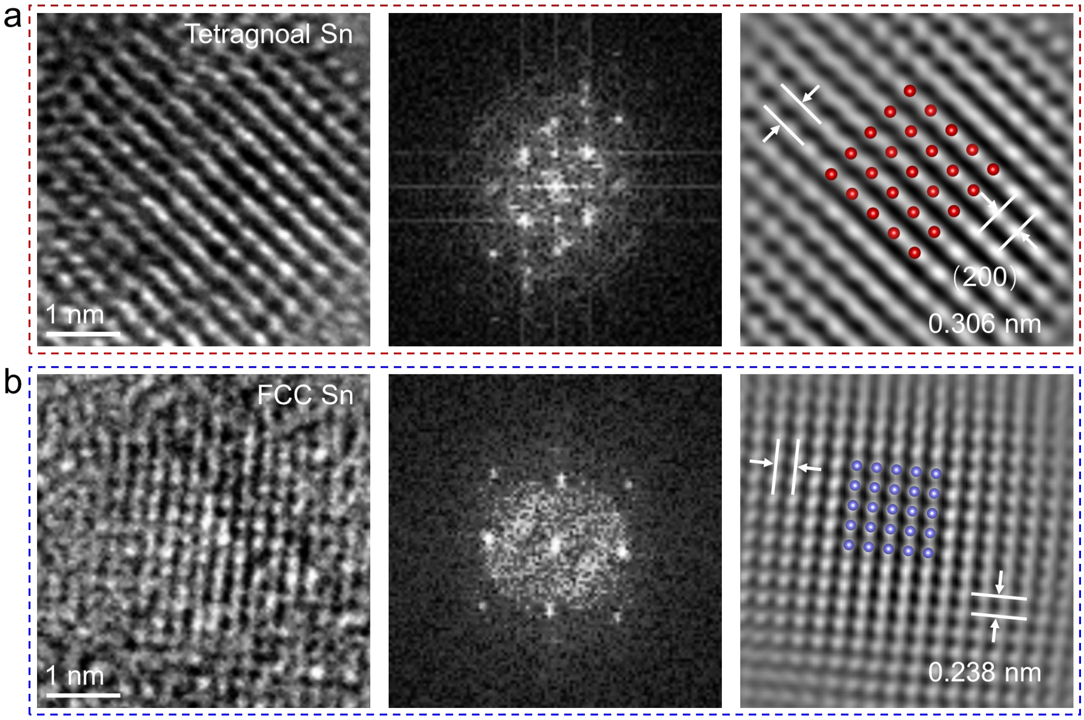

Supplementary Fig. S28. HRTEM images of tetragonal Sn (a) and FCC Sn (b) and their FFT patterns.

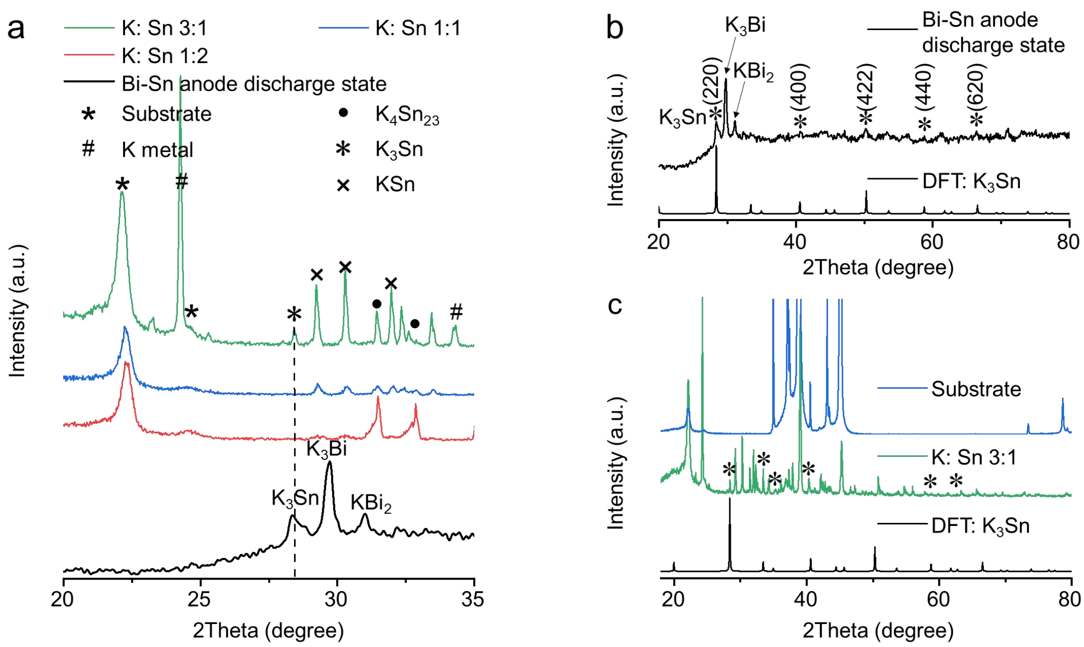

Supplementary Fig. S29. XRD tests. (a) XRD pattern of K-Sn alloys (prepared by heating and mixing K metal and Sn powder, see details for Methods) with different ratios and comparison with Bi-Sn anode in the fully discharged state (black line). (b) The simulated K_3Sn XRD pattern (theoretical calculation) compared to the experimentally measured XRD pattern of the Bi-Sn anode in the fully discharged state. (c) The simulated K_3Sn XRD pattern (black line) compared to the experimental synthesis K_3Sn XRD pattern (green line). The XRD pattern for the plastic film substrate (blue line) holder is also shown, and the peaks marked by '*' correspond to K_3Sn.

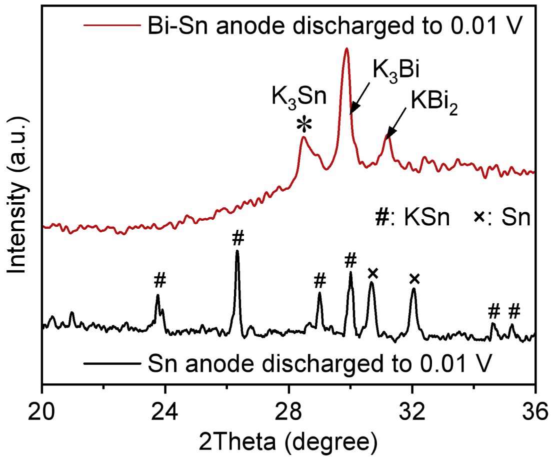

Supplementary Fig. S30. XRD pattern of pure Sn and Bi-Sn anodes in the fully discharged state.

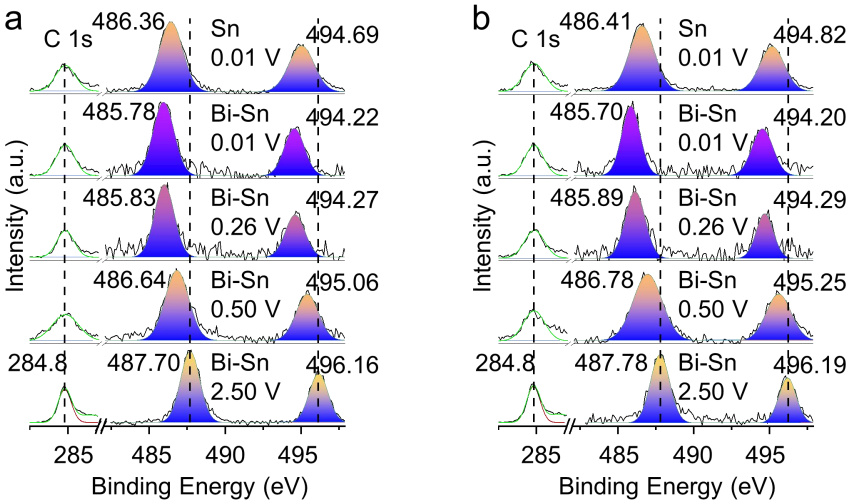

Supplementary Fig. S31. XPS Sn3d spectra of Sn (at the cut-off voltage of 0.01 V) compared to that of Bi-Sn anodes at different discharge states of the 5th (a) and 6th (b) cycles.

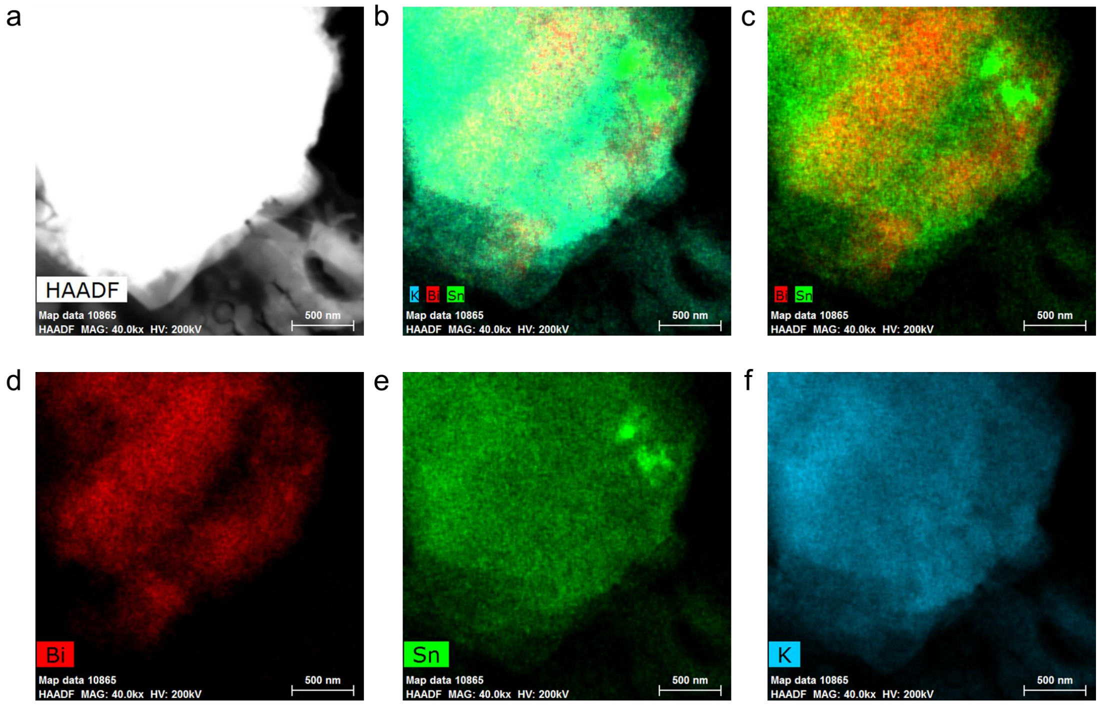

Supplementary Fig. S32. HAADF-EDS of Bi-Sn anode in the discharged state. (a) HAADF-STEM of Bi-Sn anode. The elemental distribution of K, Bi, Sn (b); Bi, Sn (c); Bi (d); Sn (e); and K (f).

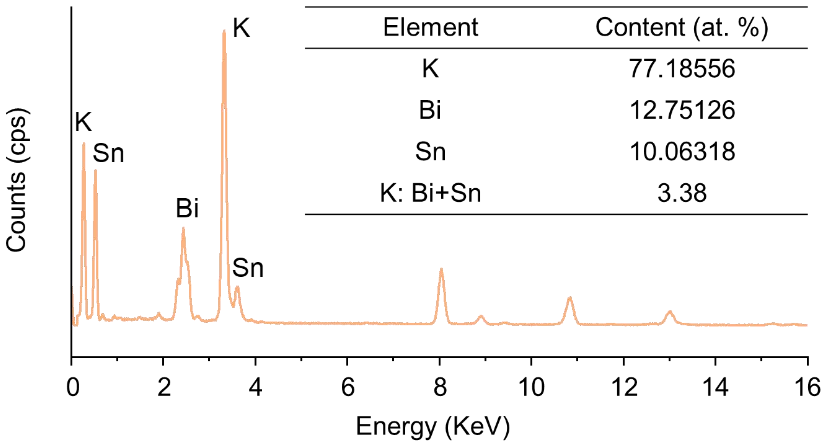

Element	Content (at. %)
K	77.18556
Bi	12.75126
Sn	10.06318
K: Bi+Sn	3.38

Supplementary Fig. S33. EDS mapping analysis. Elements in the discharge anode with a cut-off voltage of 0.01 V.

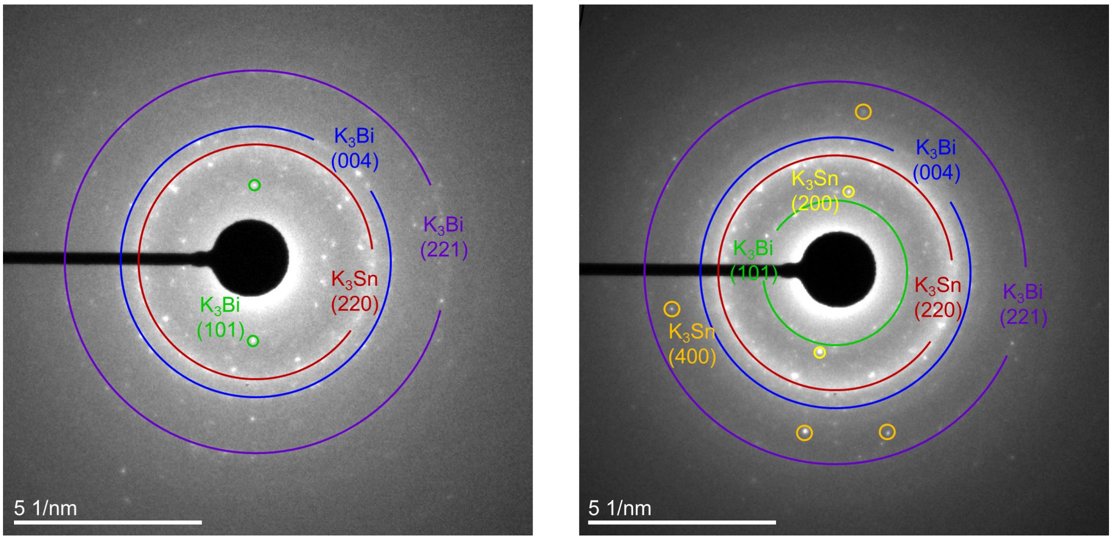

Supplementary Fig. S34. The SAED pattern of Bi-Sn anode in the discharged state.

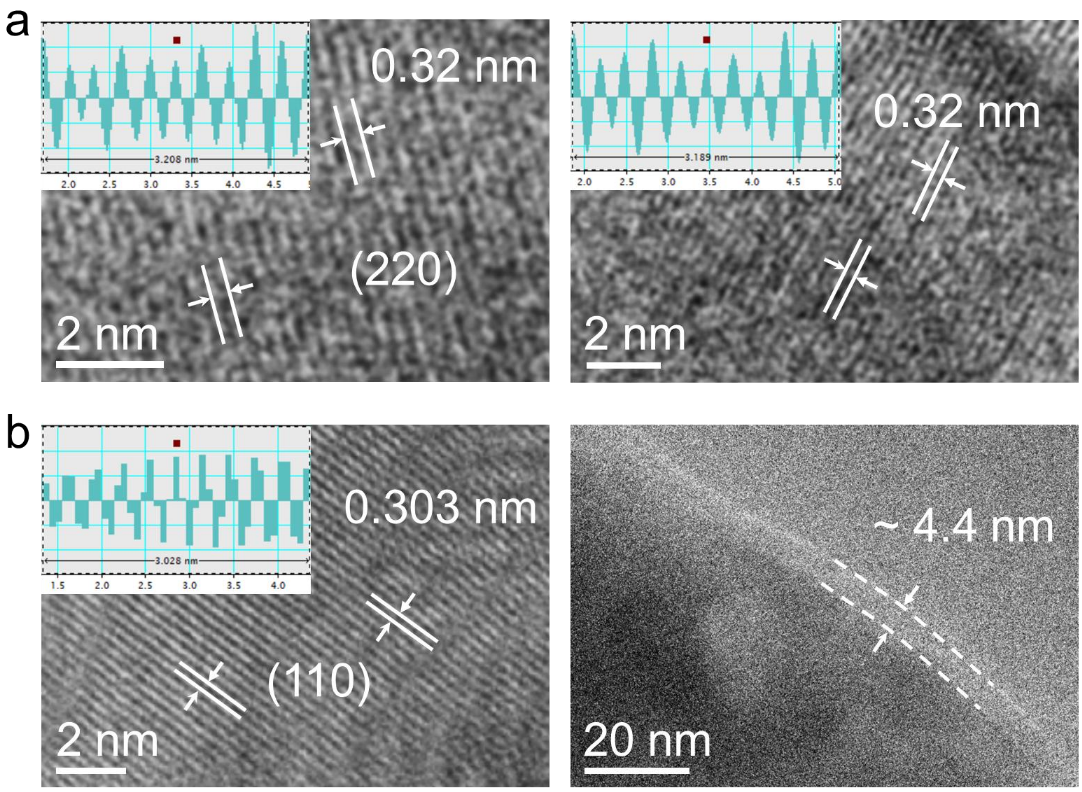

Supplementary Fig. S35. (a) Long range-ordered K_3Sn lattice (inset: average interspaces of a single particle by K_3Sn's Cryo-TEM diffraction). (b) Long range-ordered K_3Bi lattice and the uniform SEI layer (inset: average interspaces of a single particle by K_3Bi's Cryo-TEM diffraction).

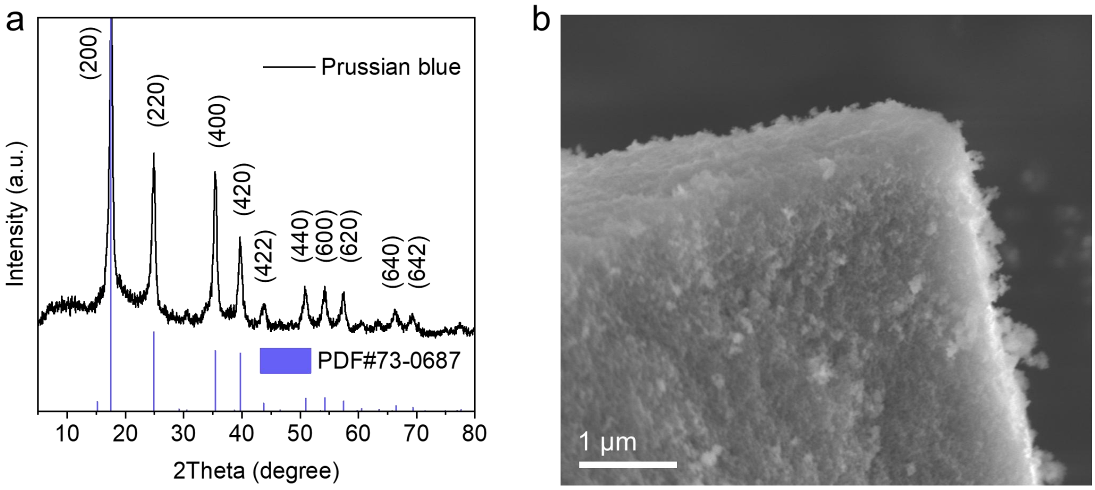

Supplementary Fig. S36. The XRD pattern of PB samples (a) and its SEM image (b).

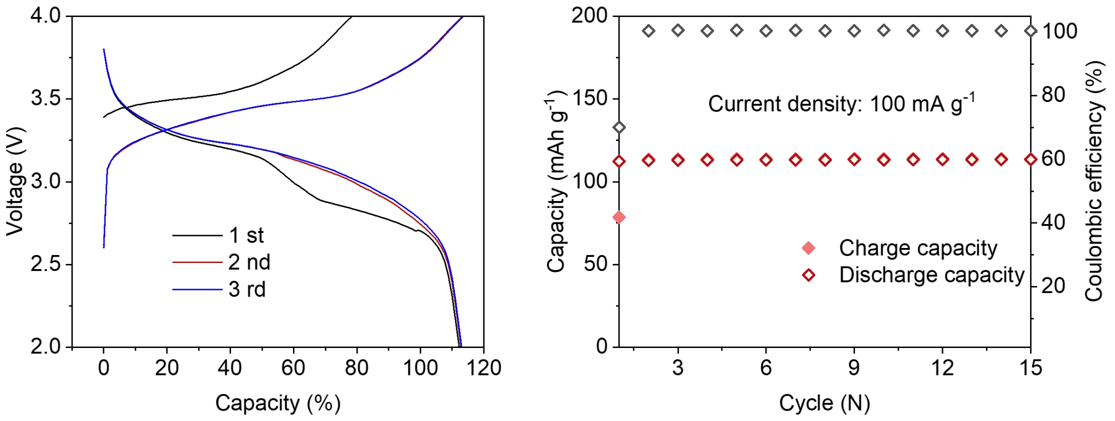

Supplementary Fig. S37. The charge/discharge curves and cycling of the K||PB half-cell.

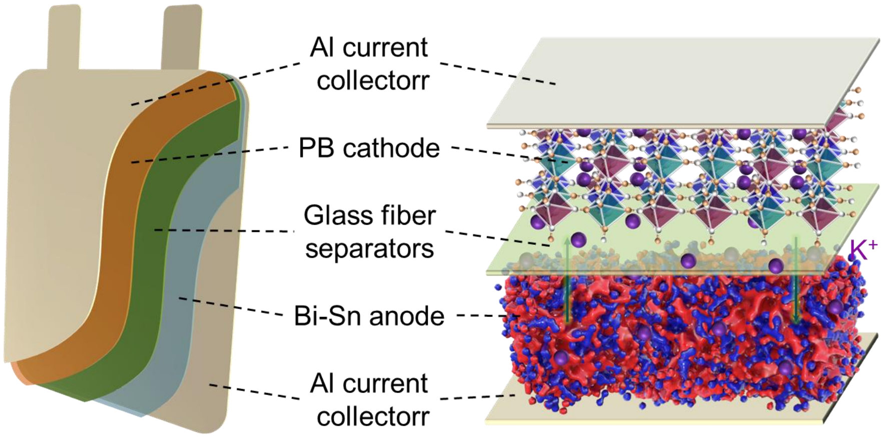

Supplementary Fig. S38. Schematic diagram of the battery structure. The soft pack and button cell construction components and the working mechanism during charge/discharge (purple: K^+).

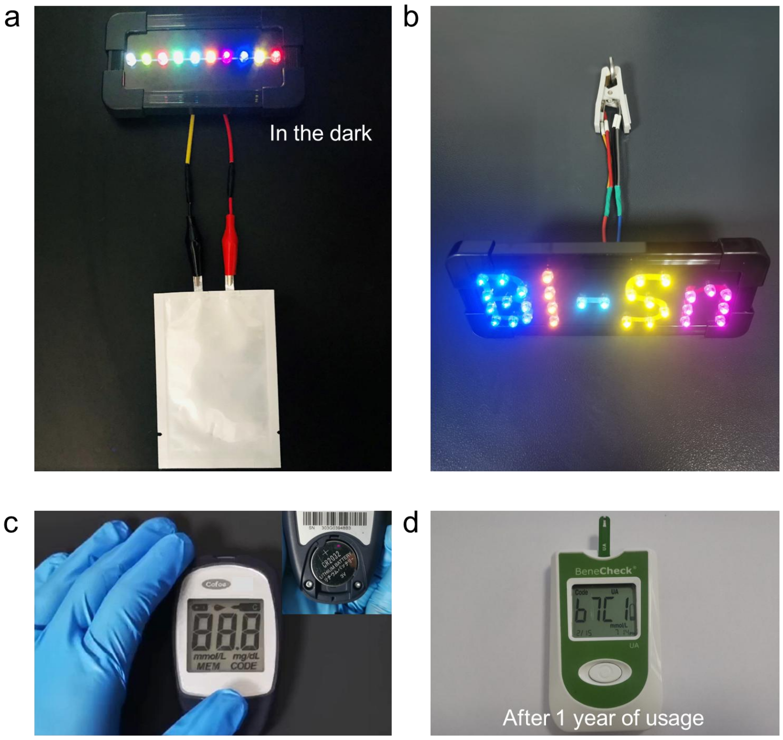

Supplementary Fig. S39. The practical applications of the designed full cells. (a) A string of lighted LEDs powered by a soft pack cell. A CR2032 battery-lighted LED panel (b) and used in blood glucose (c) or uric acid analyzer (d).

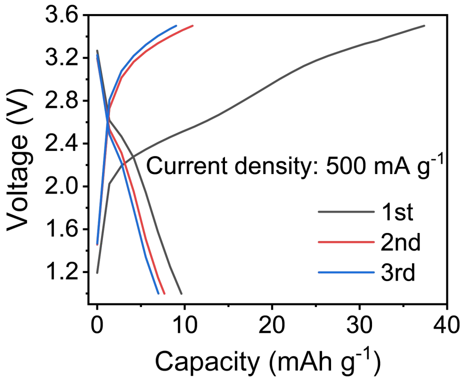

Supplementary Fig. S40. The charge-discharge curves of the full cell consisting of untreated Bi-Sn anode and PB cathode at current densities of 500 mA g^{-1}.

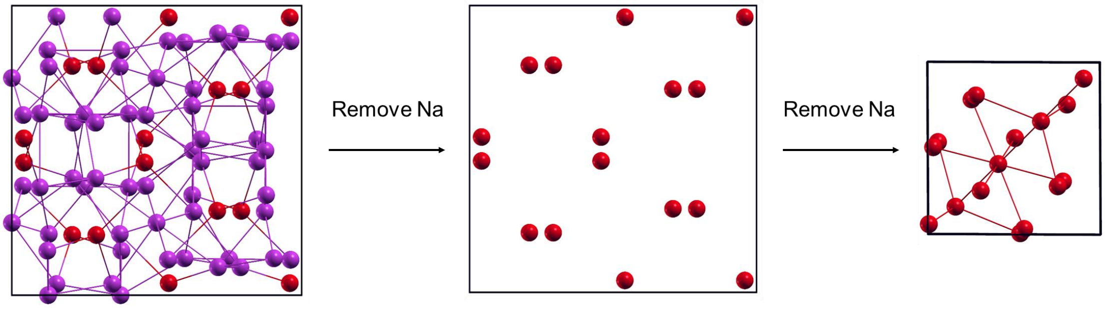

Supplementary Fig. S41. Schematic illustrations of the $Na_{15}Sn_4$ bulk cell, Na-removed Sn cell, and its optimized structure (red: Sn atom; rose: Na atom).

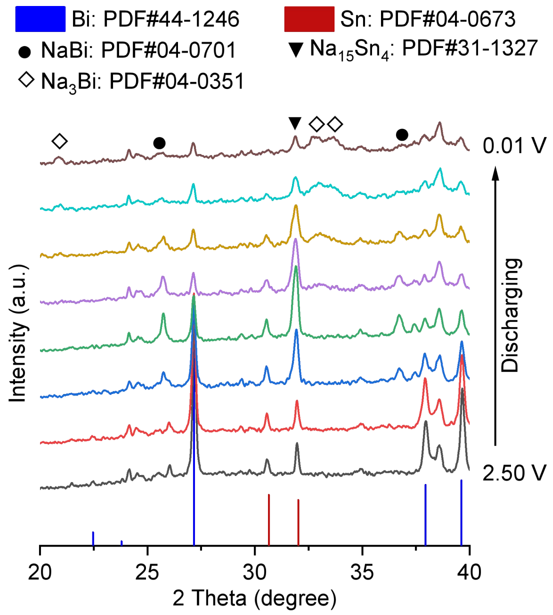

Supplementary Fig. S42. The 1D XRD scans of the Bi-Sn sodium anode (cf. Fig. 5f).

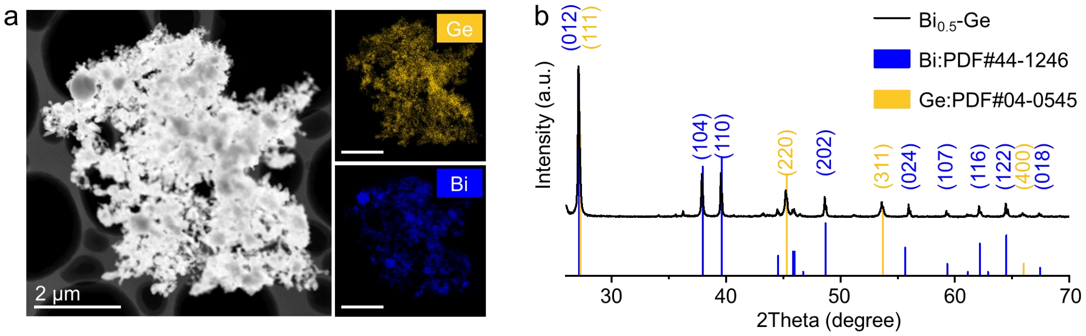

Supplementary Fig. S43. Visual structural characterization of $Bi_{0.5}$-Ge anodes. (a) HAADF-STEM and EDS mapping of $Bi_{0.5}$-Ge samples (all scale bars=2 μm). (b) XRD pattern of $Bi_{0.5}$-Ge samples.

.

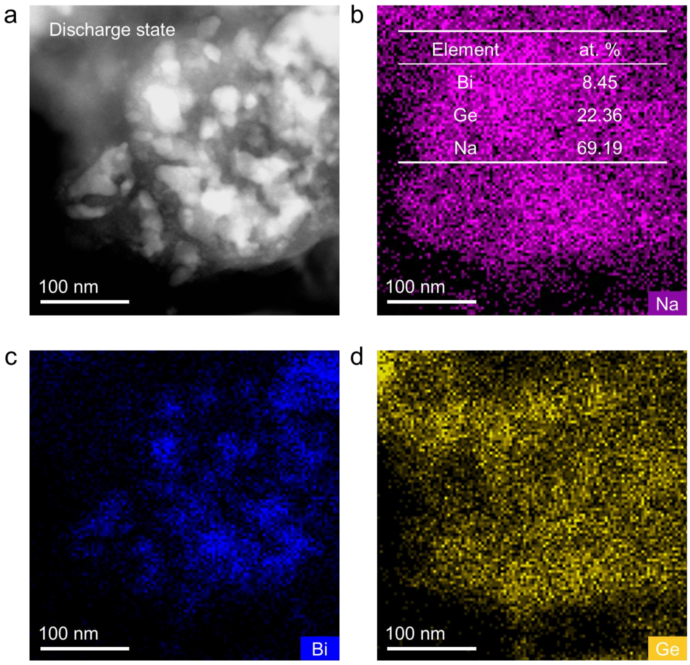

Supplementary Fig. S44. HAADF-STEM and EDS mapping of $Bi_{0.5}$-Ge sodium anode in the discharged state.

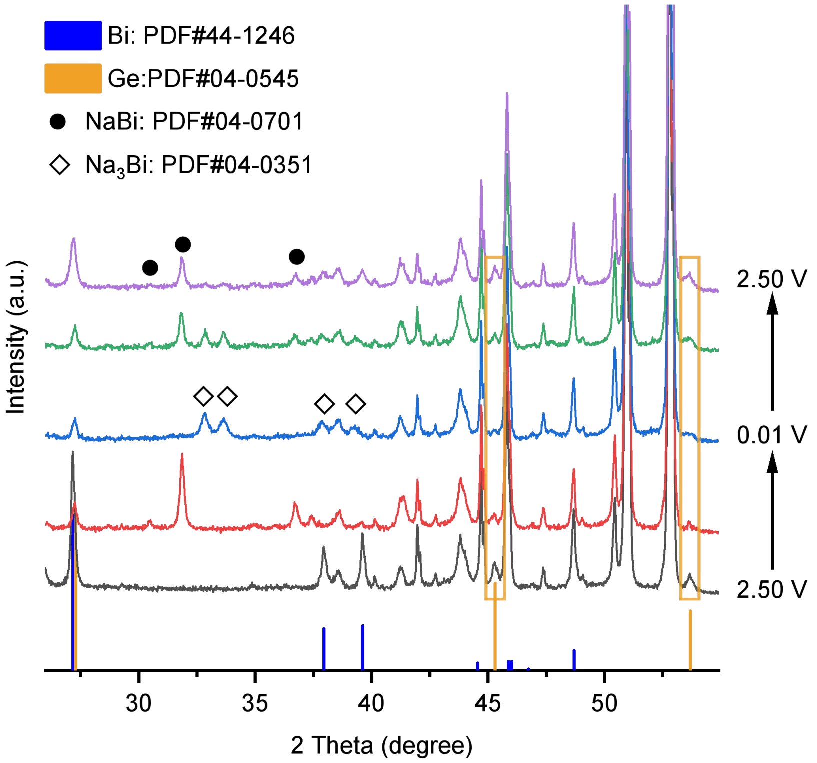

Supplementary Fig. S45. The 1D scans from *in situ* XRD study of the $Bi_{0.5}$-Ge anode sodium storage.

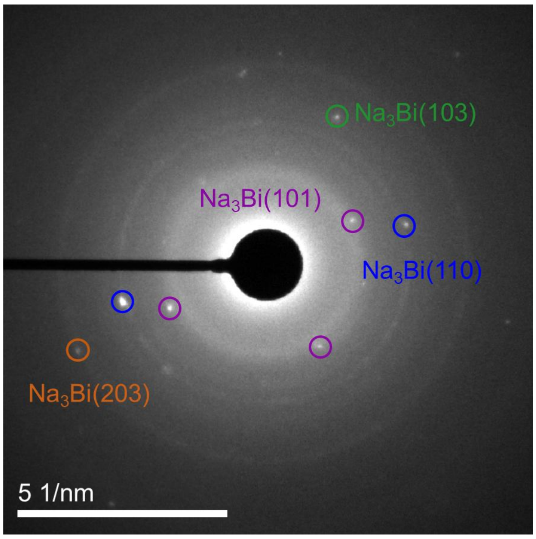

Supplementary Fig. S46. The SAED pattern of the $Bi_{0.5}$-Ge anode in the discharged state.

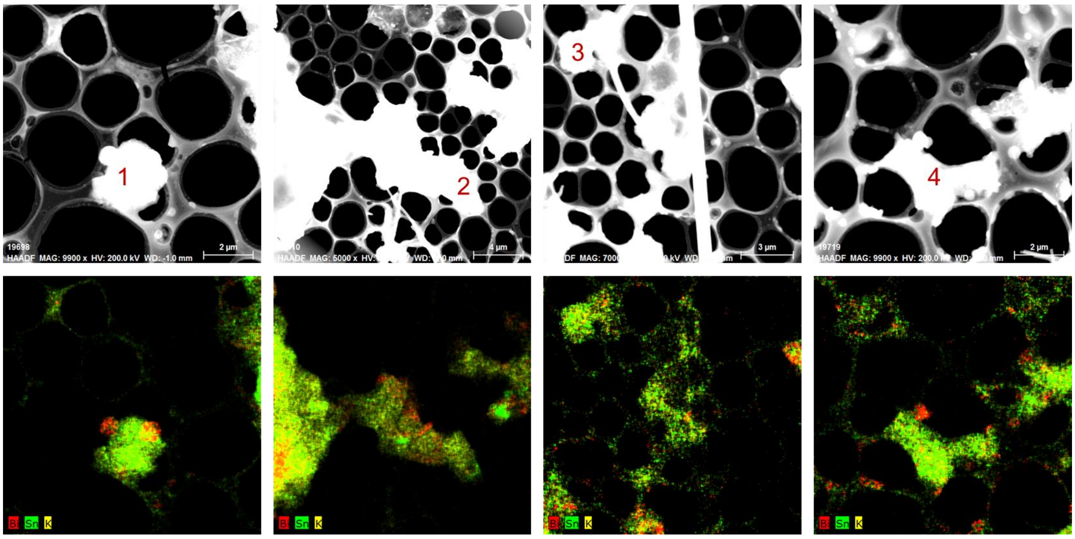

Supplementary Fig. S47. HAADF images and the EDS mapping of four target regions.

.

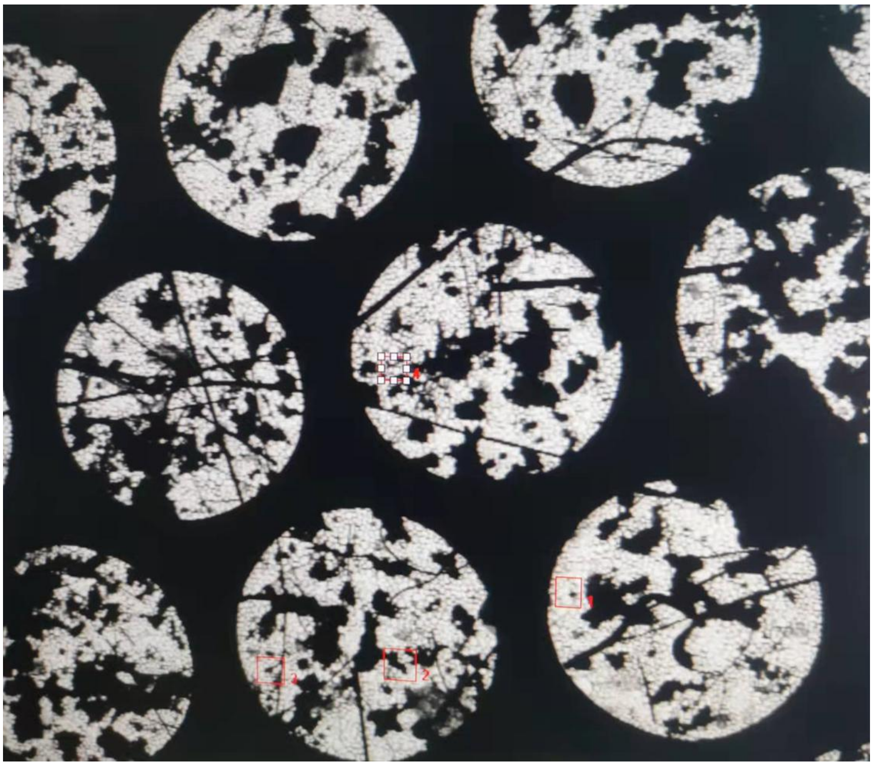

Supplementary Fig. S48. The camera recorded the exact location of the target area.

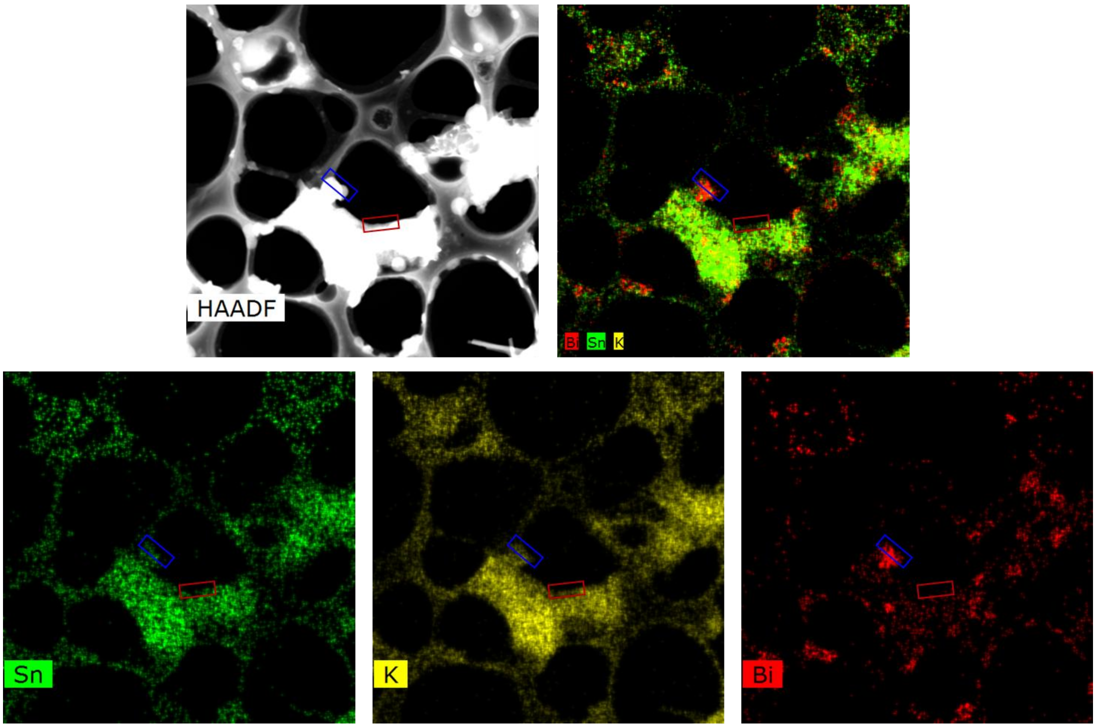

Supplementary Fig. S49. The K_3Sn and K_3Bi lattices, and the SEI on their surfaces were imaged from the red and blue frames in target area 4.

Supplementary Tables

Supplementary Table S1. The electrochemical performance of carbon, alloys, organics, oxides, and chalcogenides-based anodes in PIBs.

Name	Voltage (V)	Capacity (mAh g^{-1})	Reference
**VK@GNT**	1.0	313.5	[7]
**K_2TP**	0.575	261	[8]
**$K_2C_6O_6$**	1.375	60	[9]
**K_2SBDC@GR**	0.85	132	[10]
**$K_2C_6H_6O_4$**	1.375	207	[11]
**H_2TP**	0.525	280	[12]
**K_4PM**	0.6	292	[13]
**$K_2Ti_8O_{17}$**	0.975	180	[14]
**$K_2Ti_4O_9$**	0.625	80	[15]
**$MnCo_2O_4$**	0.825	243	[16]
**$K_2Ti_2O_5$**	0.75	150	[17]
**$K_2Ti_6O_{13}$**	0.9	100	[18]
**Graphite**	0.2	255	[19]
**CDs@rGO**	0.375	310	[20]
**$NFSC_2$**	0.6	277	[21]
**NCNFs-650**	0.65	320	[22]
**CQDHC**	0.525	265	[23]
**SC**	0.5	258	[24]
**MCUF**	0.725	343	[24]
**p-HNCs**	0.85	250	[25]
**HC**	0.3	246	[26]
**Soft carbon**	0.225	246.2	[27]
**MoSSe**	1.225	517.4	[28]
**SnS_2@C**	0.775	508	[29]

**VSe_2**	1	366	[30]
**$FeTe_2$-C**	1.25	342	[31]
**CoPSe/NC**	1.4	518	[32]
**$BiPS_4$**	0.9	554	[33]
**CuS-C@Nb_2O_5-CNFs**	1.275	278.5	[34]
**$NiSe_2$**	1.35	550	[35]
**NiS_2@C@C**	1.5	659.8	[36]
**ReS_2/N-CNFs**	1.625	350	[37]
**CoS@C**	1.25	434.5	[38]
**$CoSe_2$@NC**	1.075	450	[39]
**G@Y-S FeS_2@C**	1.25	489	[40]
**ED-MoS_2@CT**	1.0	341	[41]
**SnS_2@C-1**	1.25	457	[42]
**Sn_4P_3/C**	0.525	384.8	[43]
**Bi**	0.5	371.4	[3]
**NPCuBi**	0.45	462	[44]
**Sb**	0.5	555.7	[45]
**3D-HPCS-850**	0.6	310	[46]
**FBNs**	0.45	423	[47]
**SnP**	0.5	478.1	[48]
**Sn-Sb**	0.65	460	[49]
**BP-C**	0.65	420	[50]
**Sb@CSN**	0.55	610	[51]
**P@CN**	0.5	696.5	[52]
**BiSb@C**	0.625	420	[53]
**Sn_4P_3**	0.6	530	[54]
**K metal**	-0.09	687	[55]

Supplementary Table S2. Structural parameters of Bi-Sn based on the Rietveld refinement.

Sample	Phase name	Space Group	Lattice Parameters	Reliability Parameters
**Bi-Sn**	**Bi structure**	R $\bar{3}$ m [166]	a=b=4.54 Å<br>c=11.87 Å<br>α=90°<br>β=90°<br>γ=120°	Rwp=9.990%<br>Rp=7.221%<br>Rexp=7.363%<br>Gof=1.357
	**Sn structure**	$I4_1$/amd [141]	a=b=5.84 Å<br>c=3.19 Å<br>α=90°<br>β=90°<br>γ=90°	

Supplementary Table S3. ICP test results were compared with experimental results.

Name	Preset mole ratio	Element	Experimental mass (g)	ICP test mass fraction (%)	Actual mole ratio
**Bi-Sn**	**0.5:1**	Bi	0.273	48.2947	0.53:1
		Sn	0.254	51.0984	
	**1:1**	Bi	0.546	64.0490	1.03:1
		Sn	0.254	35.2631	
	**1.5:1**	Bi	0.825	74.4763	1.68:1
		Sn	0.256	25.1734	

Supplementary Table S4. Rate capacities for five anodes.

	Name	Current density (mA g^{-1})						Capacity retention (%)	Capacity recovery (%)
		50	100	200	500	…	50		
Reversible capacity (mAh g^{-1})	**Sn**	190	83	35	12	…	98	6.3%	52%
	**Bi**	369	256	107	12	…	214	3.3%	58%
	**$Bi_{0.5}$-Sn**	414	362	327	255	…	310	62%	75%
	**$Bi_{1.5}$-Sn**	496	449	417	352	…	423	71%	85%
	**Bi-Sn**	612	572	542	484	…	589	79%	96%

Supplementary Table S5. Electrochemical properties of alloy anodes in PIB.

Name	Voltage (V)	Cycle (N)	Capacity (mAh g^{-1})	Reference
**Bi@C**	0.55	20	193.6	[56]
**Sb_2MoO_6**	0.75	50	381	[57]
**Bi/rGO**	0.525	50	290	[58]
**Sn_4P_3/C**	0.35	50	307.2	[59]
**NP-Sb-20**	0.475	50	318	[60]
**Bi_2MoO_6**	0.4	50	208.9	[61]
**Sn-C**	0.675	30	110	[62]
**Sn@RGO**	0.75	50	200	[63]
**NPCuBi**	0.45	50	462	[44]
**NiSn@C**	0.75	300	322.4	[64]
**Sb**	0.55	180	555.7	[45]
**Sb@CSN**	0.55	220	504	[65]
**Sb@CNFs**	0.625	200	338	[66]
**Sn/NPC**	0.875	200	198	[67]
**$SnP_{0.94}$@GO**	0.65	100	106	[68]
**SnSb@MAC**	1	100	312	[69]
**BCO**	0.845	120	430	[70]
**3D-HPCS-650**	0.7	100	276.4	[71]
**SnP**	0.5	100	478.1	[48]
**Sn_4P_3**	0.6	200	530	[54]
**Bi-Sn**	0.35	500	634	This Work

Supplementary Table S6. ICP test results of potassium storage in Bi-Sn anode in the discharge state.

Elemental content (at. %)	Number 1	Number 2	Number 3
**Bi**	0.363793	0.342365	0.338641
**Sn**	0.358400	0.351009	0.348100
**K**	2.357480	2.336975	2.281350
**Ratio (K: Bi+Sn)**	3.26	3.37	3.32

Supplementary Table S7. ICP results of sodium storage in the $Bi_{0.5}$-Ge anode in the discharged state.

Elemental content (at. %)	Number 1	Number 2	Number 3
**Bi**	1.126647	1.008996	0.908650
**Ge**	2.032033	1.919999	2.024323
**Na**	7.350587	7.096427	7.490631

Supplementary References

1. Garrity KF, Bennett JW and Rabe KM *et al.* Pseudopotentials for high-throughput DFT calculations. *Comp Mater Sci* 2014; **81**: 446-52.
2. Giannozzi P, Baroni S and Bonini N *et al.* QUANTUM ESPRESSO: a modular and open-source software project for quantum simulations of materials. *J Phys Condens Matter* 2009; **21**: 395502.
3. Lei K, Wang C and Liu L *et al.* A porous network of bismuth used as the anode material for high-energy-density potassium-ion batteries. *Angew Chem Int Ed* 2018; **57**: 4687-91.
4. Huang J, Lin X and Tan H *et al.* Bismuth microparticles as advanced anodes for potassium-ion battery. *Adv Energy Mater* 2018; **8**: 1703496.
5. Qu J, Xiao J and Wang T *et al.* High rate transfer mechanism of lithium ions in lithium-tin and lithium-indium alloys for lithium batteries. *J Phys Chem C* 2020; **124**: 24644-52.
6. Lv Y, Tong Q and Liu Y *et al.* Band-offset degradation in van der waals heterojunctions. *Phys Rev Appl* 2019; **12**: 044064.
7. Xue Q, Li D and Huang Y *et al.* Vitamin K as a high-performance organic anode material for rechargeable potassium ion batteries. *J Mater Chem A* 2018; **6**: 12559-64.
8. Lei K, Li F and Mu C *et al.* High K-storage performance based on the synergy of dipotassium terephthalate and ether-based electrolytes. *Energy Environ Sci* 2017; **10**: 552-57.
9. Zhao Q, Wang J and Lu Y *et al.* Oxocarbon salts for fast rechargeable batteries. *Angew Chem Int Ed* 2016; **55**: 12528-32.
10. Li C, Deng Q and Tan H *et al.* Para-conjugated dicarboxylates with extended aromatic skeletons as the highly advanced organic anodes for K-ion battery. *ACS Appl Mater Interfaces* 2017; **9**: 27414-20.
11. Deng Q, Tian C and Luo Z *et al.* Organic 2,5-dihydroxy-1,4-benzoquinone potassium salt with ultrahigh initial coulombic efficiency for potassium-ion batteries *Chem Commun* 2020; **56**: 12234-37.
12. Wang C, Tang W and Yao Z *et al.* Using an organic acid as a universal anode for highly efficient Li-ion, Na-ion and K-ion batteries. *Org Electron* 2018; **62**: 536-41.
13. Pan Q, Zheng Y and Tong Z *et al.* Novel lamellar tetrapotassium pyromellitic organic for robust high-capacity potassium storage. *Angew Chem Int Ed* 2021; **60**: 11835-40.
14. Han J, Xu M and Niu Y *et al.* Exploration of $K_2Ti_8O_{17}$ as an anode material for potassium-ion batteries. *Chem Commun* 2016; **52**: 11274-76.
15. Kishore B, G V and Munichandraiah N. $K_2Ti_4O_9$: A promising anode material for potassium ion batteries. *J Electrochem Soc* 2016; **163**: A2551.
16. Huang R, Lin J and Zhou J *et al.* Hierarchical triple-shelled $MnCo_2O_4$ hollow microspheres as high-performance anode materials for potassium-ion batteries. *Small* 2021; **17**: e2007597.
17. Zhao S, Dong L and Sun B *et al.* $K_2Ti_2O_5$@C microspheres with enhanced K^+

intercalation pseudocapacitance ensuring fast potassium storage and long-term cycling stability. *Small* 2020; **16**: e1906131.

18. Li D, Liu C and Huang D *et al.* Atomic layer deposition regulating hydrated $K_2Ti_6O_{13}$ nanobelts on graphene platform with accelerated solid solution potassiation for potassium ion capacitors. *Chem Eng J* 2021; **417**: 128048.
19. Fan L, Ma R and Zhang Q *et al.* Graphite anode for a potassium-ion battery with unprecedented performance. *Angew Chem Int Ed* 2019; **58**: 10500-05.
20. Zhang E, Jia X and Wang B *et al.* Carbon dots@rGO paper as freestanding and flexible potassium-ion batteries anode. *Adv Sci* 2020; **7**: 2000470.
21. Zhong Y, Dai W and Liu D *et al.* Nitrogen and fluorine dual doping of soft carbon nanofibers as advanced anode for potassium ion batteries. *Small* 2021; **17**: e2101576.
22. Xu Y, Zhang C and Zhou M *et al.* Highly nitrogen doped carbon nanofibers with superior rate capability and cyclability for potassium ion batteries. *Nat Commun* 2018; **9**: 1720.
23. Guo Y, Feng Y and Li H *et al.* Carbon quantum dots in hard carbon: an approach to achieving PIB anodes with high potassium adsorption. *Carbon* 2022; **189**: 142-51.
24. Liu H, Du H and Zhao W *et al.* Fast potassium migration in mesoporous carbon with ultrathin framework boosting superior rate performance for high-power potassium storage. *Energy Storage Materials* 2021; **40**: 490-98.
25. Hong W, Zhang Y and Yang L *et al.* Carbon quantum dot micelles tailored hollow carbon anode for fast potassium and sodium storage. *Nano Energy* 2019; **65**: 104038.
26. Alvin S, Cahyadi H and Hwang J *et al.* Revealing the intercalation mechanism of lithium, sodium and poassium in hard carbon. *Adv Energy Mater* 2020; **10**: 2000283.
27. Liu Y, Lu Y and Xu Y *et al.* Pitch-derived soft carbon as stable anode material for potassium ion batteries. *Adv Mater* 2020; **32**: e2000505.
28. He H, Huang D and Gan Q *et al.* Anion vacancies regulating endows MoSSe with fast and stable potassium ion storage. *ACS Nano* 2019; **13**: 11843-52.
29. Sun Q, Li D and Dai L *et al.* Structural engineering of SnS_2 encapsulated in carbon nanoboxes for high-performance sodium/potassium-ion batteries anodes. *Small* 2020; **16**: e2005023.
30. Yang C, Feng J and Lv F *et al.* Metallic graphene-like VSe_2 ultrathin nanosheets: superior potassium-ion storage and their working mechanism. *Adv Mater* 2018; **30**: e1800036.
31. Park G, Kang Y. Conversion reaction mechanism for yolk-shell-structured iron telluride-C nanospheres and exploration of their electrochemical performance as an anode material for potassium-ion batteries. *Small Methods* 2020; **4**: 2000556.
32. Feng Y, Xu M and He T *et al.* CoPSe: a new ternary anode material for stable and high-rate sodium/potassium-ion batteries. *Adv Mater* 2021; **33**: e2007262.
33. Haghighat-Shishavan S, Nazarian-Samani M and Nazarian-Samani M *et al.* Electrolyte modulation of $BiPS_4$ concurrently suppressing the Bi coarsening and

polysulfide shuttle effect in K-ion batteries. *Energy Storage Materials* 2021; **39**: 96-107.
34. Cao K, Zheng R and Wang S *et al.* Boosting coulombic efficiency of conversion-reaction anodes for potassium-ion batteries via confinement effect. *Adv Funct Mater* 2020; **30**: 2007712.
35. Chu J, Yu Q and Han K *et al.* A novel graphene-wrapped corals-like $NiSe_2$ for ultrahigh-capacity potassium ion storage. *Carbon* 2020; **161**: 834-41.
36. Yang L, Hong W and Zhang Y *et al.* Hierarchical NiS_2 modified with bifunctional carbon for enhanced potassium-ion storage. *Adv Funct Mater* 2019; **29**: 1903454.
37. Mao M, Cui C and Wu M *et al.* Flexible ReS_2 nanosheets/N-doped carbon nanofibers-based paper as a universal anode for alkali (Li, Na, K) ion battery. *Nano Energy* 2018; **45**: 346-52.
38. Gao H, Zhou T and Zheng Y *et al.* CoS quantum dot nanoclusters for high-energy potassium-ion batteries. *Adv Funct Mater* 2017; **27**: 1702634.
39. Hu J, Wang B and Yu Q *et al.* $CoSe_2$/N-doped carbon porous nanoframe as an anode material for potassium-ion storage. *Nanotechnology* 2020; **31**: 395403.
40. Zhao Y, Zhu J and Ong S *et al.* High-rate and ultralong cycle-life potassium ion batteries enabled by in situ engineering of yoll-shell FeS_2@C structure on graphene matrix. *Adv Energy Mater* 2018; **8**: 1802565.
41. Cui Y, Liu W and Feng W *et al.* Controlled design of well-dispersed ultrathin MoS_2 nanosheets inside hollow carbon skeleton: toward fast potassium storage by constructing spacious “houses” for K ions. *Adv Funct Mater* 2020; **30**: 1908755.
42. Li D, Dai L and Ren X *et al.* Foldable potassium-ion batteries enabled by free-standing and flexible SnS_2@C nanofibers. *Energy Environ Sci* 2021; **14**: 424.
43. Zhang W, Mao J and Li S *et al.* Phosphorus-based alloy materials for advanced potassium-ion battery anode. *J Am Chem Soc* 2017; **139**: 3316-19.
44. Wu X, Zhang W and Wu N *et al.* Exploration of nanoporous CuBi binary alloy for potassium storage. *Adv Funct Mater* 2020; **30**: 2003838.
45. Du X, Gao Y and Zhang B. Building elastic solid electrolyte interphases for stabilizing microsized antimony anodes in potassium ion batteries. *Adv Funct Mater* 2021; **31**: 2102562.
46. Huang K, Xing Z and Wang L *et al.* Direct synthesis of 3D hierarchically porous carbon/Sn composites via in situ generated NaCl crystals as templates for potassium-ion batteries anode. *J Mater Chem A* 2018; **6**: 434-42.
47. Shen C, Cheng T and Liu C *et al.* Bismuthene from sonoelectrochemistry as a superior anode for potassium-ion batteries. *J Mater Chem A* 2020; **8**: 453-60.
48. Li B, Shang S and Zhao J *et al.* Metastable trigonal SnP: A promising anode materrial for potassium-ion battery. *Carbon* 2020; **168**: 468-74.
49. Ding H, Wang J and Fan L *et al.* Sn-Sb coumpounds with novel structure for stable potassium strorage. *Chem Eng J* 2020; **395**: 125147.
50. Sultana I, Rahman M and Ramireddy T *et al.* High capacity potassium-ion

battery anodes based on black phosphorus. *J Mater Chem A* 2017; **5**: 23506.
51. Zheng J, Yang Y and Fan X *et al.* Extremely stable antimony-carbon composite anodes for potassium-ion batteries. *Energy Environ Sci* 2019; **12**: 615.
52. Xiong P, Bai P and Tu S *et al.* Red phosphorus nanoparticle@3D interconnected carbon nanosheet framework composite for potassium-ion battery anodes. *Small* 2018; **14**: 1802140.
53. Xiong P, Wu J and Zhou M *et al.* Bismuth-antimony alloy nanoparticle@porous carbon nanosheet composite anode for high-performance potassium-ion batteries. *ACS Nano* 2020; **14**: 1018-26.
54. Zhang W, Pang W and Sencadas V *et al.* Understanding high-energy-density Sn_4P_3 anodes for potassium-ion batteries. *Joule* 2018; **2**: 1534-47.
55. Liu P, Wang Y and Gu Q *et al.* Dendrite-free potassium metal anodes in a carbonate electrolyte. *Adv Mater* 2020; **32**: e1906735.
56. Zhang R, Bao J and Wang Y *et al.* Concentrated electrolytes stabilize bismuth-potassium batteries. *Chem Sci* 2018; **9**: 6193-98.
57. Wang J, Wang B and Liu Z *et al.* Nature of bimetallic oxide Sb_2MoO_6/rGO anode for high-performance potassium-ion batteries. *Adv Sci* 2019; **6**: 1900904.
58. Zhang Q, Mao J and Pang W *et al.* Boosting the potassium storage performance of alloy-based anode materials via electrolyte salt chemistry. *Adv Energy Mater* 2018; **8**: 1703288.
59. Zhang W, Mao J and Li S *et al.* Phosphorus-based alloy materials for advanced potassium-ion battery anode. *J Am Chem Soc* 2017; **139**: 3316-19.
60. An Y, Tian Y and Ci L *et al.* Micron-sized nanoporous antimony with tunable porosity for high-performance potassium-ion batteries. *ACS Nano* 2018; **12**: 12932-40.
61. Hu J, Xie Y and Zheng J *et al.* Unveiling nanoplates-assembled Bi_2MoO_6 microsphere as a novel anode material for high performance potassium-ion batteries. *Nano Res* 2020; **13**: 2650-57.
62. Sultana I, Ramireddy T and Rahman M *et al.* Tin-based composite anodes for potassium-ion batteries. *Chem Commun* 2016; **52**: 9279-82.
63. Wang H, Xing Z and Hu Z *et al.* Sn-based submicron-particles encapsulated in porous reduced graphene oxide network: advanced anodes for high-rate and long life potassium-ion batteries. *Appl Mater Today* 2019; **15**: 58-66.
64. Li J, Xu X and Yu X *et al.* Monodisperse CoSn and NiSn nanoparticles supported on commercial carbon as anode for lithium- and potassium-ion batteries. *ACS Appl Mater Interfaces* 2020; **12**: 4414-22.
65. Zheng J, Yang Y and Fan X *et al.* Extremely stable antimony-carbon composite anodes for potassium-ion batteries. *Energy Environ. Sci.* 2019; **12**: 615.
66. Huang H, Wang J and Yang X *et al.* Unveiling the advances of nanostructure design for alloy-type potassium-ion battery anodes via in situ TEM. *Angew Chem Int Ed* 2020; **59**: 14504-10.
67. Yang Y, Li D and Zhang J *et al.* Sn nanoparticles anchored on N doped porous carbon as an anode for potassium ion batteries. *Mater Lett* 2019; **256**: 126613.
68. Zhao X, Wang W and Hou Z *et al.* $SnP_{0.94}$ nanoplates/graphene oxide composite

for novel potassium-ion battery anode. *Chem Eng J* 2019; **370**: 677-83.
69. Hsieh Y, Chen K and Tuan H. A synergetic Sn-Sb amorphous carbon composites compared from polyesterification process as an ultrastable potassium-ion batttery. *Chem Eng J* 2021; **420**: 130451.
70. Wang L, Zhang B and Wang B *et al.* In-situ nano-crystallization and solvation modulation to promote highly stable anode involving alloy/de-alloy for potassium ion batteries. *Angew Chem Int Ed* 2021; **60**: 15381-89.
71. Huang K, Xing Z and Wang L *et al.* Direct synthesis of 3D hierarchically porous carbon/Sn composites via in situ generated NaCl crystals as templates for potassium-ion batteries anode. *J Mater Chem A* 2018; **6**: 434-42.
